# Robust temporal pumping in a magneto-mechanical topological insulator

**DOI:** 10.1038/s41467-020-14804-0

**Published:** 2020-02-20

**Authors:** Inbar Hotzen Grinberg, Mao Lin, Cameron Harris, Wladimir A. Benalcazar, Christopher W. Peterson, Taylor L. Hughes, Gaurav Bahl

**Affiliations:** 1 Department of Mechanical Science and Engineering, University of Illinois at Urbana-Champaign, Urbana, IL 61801 USA; 2 Department of Physics, University of Illinois at Urbana-Champaign, Urbana, IL 61801 USA; 3 Department of Electrical and Computer Engineering, University of Illinois at Urbana-Champaign, Urbana, IL 61801 USA

**Keywords:** Metamaterials, Mechanical properties, Topological insulators

## Abstract

The transport of energy through 1-dimensional (1D) waveguiding channels can be affected by sub-wavelength disorder, resulting in undesirable localization and backscattering phenomena. However, quantized disorder-resilient transport is observable in the edge currents of 2-dimensional (2D) topological band insulators with broken time-reversal symmetry. Topological pumps are able to reduce this higher-dimensional topological insulator phenomena to lower dimensionality by utilizing a pumping parameter (either space or time) as an artificial dimension. Here we demonstrate a temporal topological pump that produces on-demand, robust transport of mechanical energy using a 1D magneto-mechanical metamaterial. We experimentally demonstrate that the system is uniquely resilient to defects occurring in both space and time. Our findings open a path towards exploration of higher-dimensional topological physics with time as a synthetic dimension.

## Abstract (web summary)

A robust time-controlled energy pumping in a classical metamaterial remains to be achieved. Here, Grinberg et al. demonstrate a temporal topological pump that produces on-demand, robust transport of mechanical energy using a one-dimensional magneto-mechanical metamaterial.

## Introduction

The discovery that topological insulators host protected boundary states has spurred significant research on their metamaterial analogs due to attractive prospects in both science and engineering. A particularly important feature is the robust propagation that is observable in the chiral edge modes of 2D topological insulators having broken time-reversal symmetry, otherwise broadly known as Chern insulators^1–3^. This class of systems includes integer quantum Hall insulators^4^, the quantum anomalous Hall insulator^3,5^, and their metamaterial analogs^6–11^, all of which can produce quantized transport even with significant disorder.

In this context, it was shown that periodic, adiabatic, spatio-temporal modulations of a 1D periodic potential can also produce quantized particle transport^12^ where the number of particles pumped in one cycle is equal to the Chern number defined on the (1 + 1)-dimensional Brillouin zone spanned by momentum and time^13^. Thus, an adiabatic pumping process may be regarded as a dynamical manifestation of a Chern insulator in one higher dimension^3^, and as such is similarly topologically robust against disorder and defects^14^. Topological pumps have been implemented in a variety of systems including realization of Rice-Mele’s model^15,16^, cold atomic gases^17–20^ and classical metamaterials^21–23^. Significant explorations in photonic metamaterials include using topological pumps to map the Berry curvature^16,24^, to demonstrate transport of a localized mode in a quasiperiodic waveguide array^25,26^, and to probe a four dimensional quantum Hall effect^27^. However, to date, a temporally-controlled topological pump that produces on-demand, disorder-resilient transport has not been demonstrated in any metamaterial system.

Topological pumping can be understood as a consequence of the spectral flow property^28,29^ of topological band structure. For the conventional 1D topological pump, the band structure evolves from topologically non-trivial to trivial and back during one pumping cycle, and crucially, reflection and time-reversal symmetries are not preserved. For a system with open boundaries an integer number of eigenstates flow from a lower energy band to an upper energy band, i.e. across the bulk energy gap, during this process, and the spatial profile of the flowing modes migrates from one end of the system to the other while carrying, e.g., charge, spin, or energy. Given a pumping protocol we can calculate a well-known topological invariant called the Chern number. This invariant dictates the quantity of spectrally flowing modes, and hence the amount of, e.g., charge, spin, or energy that is robustly transported across the (meta)material during one cycle of an ideal pump.

From a tight-binding model perspective^30^, a topological pump can be produced through spatio-temporal modulation of the on-site potentials and couplings between the constitutive elements of a metamaterial platform. However, not all cyclic spatio-temporal modulations generate non-vanishing Chern numbers. Even if a protocol produces a Chern number, there are additional dynamical constraints for achieving robust transport. Namely, this process must be performed adiabatically to ensure that energy from the spectrally flowing states does not leak to the bulk bands. At the same time, since physical systems have finite loss, the topological pump must complete a pump cycle faster than the decay time of the state being transported. For photonic implementations, the latter requirement necessitates extremely rapid modulation, which is technically very challenging. To date, a workaround has been to use space instead of time as the pumping parameter^10,24–27,31^. A time-controlled classical topological pump has remained elusive to date, and as a result, on-demand robust pumping of energy in a classical metamaterial has not yet been achieved.

In this work, we demonstrate a temporal topological pump using a 1D metamaterial composed of magnetically-coupled mechanical resonators. Pumping is achieved by replicating a 2D Chern insulator in one spatial dimension and one temporal dimension. A non-contact approach is employed to produce the necessary modulations of the couplings and on-site potentials using permanent magnets and a high-permeability metal alloy mounted on a common rotating shaft. This system can, in principle, be hand cranked to pump energy on-demand, in a manner reminiscent of an Archimedes Screw. We experimentally demonstrate that mechanical energy can be robustly transported across the entire metamaterial in exactly one pumping cycle, as long as dynamical requirements listed above are met. We further demonstrate through a series of experiments that the topological pump is robust against disorder that may appear either in space or time.

## Results

### Prescription of the topological pump

**Fig. 1:**
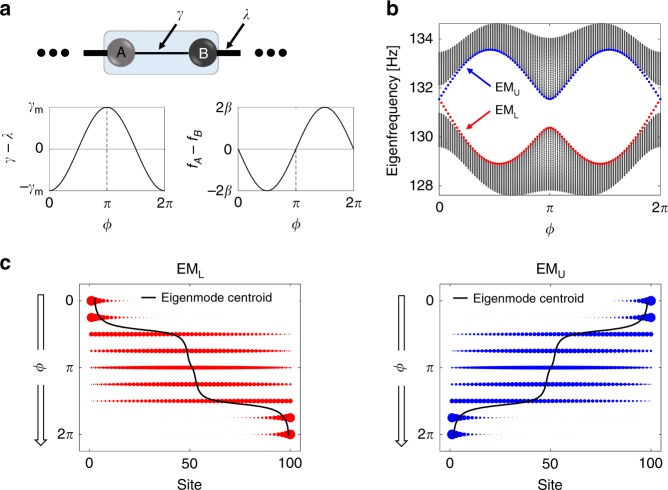
Description of the topological pump. **a** A unit cell of a dimerized 1D array (sub-lattice sites A, B) having on-site potential *f*_A_, *f*_B_, intra-cell coupling *γ* and inter-cell coupling *λ*. The pump is produced by modulating the intra-cell coupling with a $\cos (\phi )$ trend, and modulating the on-site potentials with a $\sin (\phi )$ trend, where *ϕ* ∈ [0, 2*π*] is the pump phase. **b** Calculated band-structure for an array composed of 100 sites, as a function of the pump phase *ϕ*. EM_U_ and EM_L_ are the upper and lower trajectories over which the two edge modes evolve during the pump cycle. At *ϕ* = 0, 2*π* the system is in the topologically non-trivial phase while at *ϕ* = *π* the system is in the topologically trivial phase. **c** Evolution of the EM_L_ (red) and EM_U_ (blue) eigenmodes during the pump cycle. Dots represent the magnitude of the eigenmode at each site. EM_L_ is localized on the left edge at *ϕ* = 0 and transports to the right edge. In contrast, EM_U_ is transported from the right edge to the left edge. The solid black line represents the centroid of the eigenmode calculated through ∑_*i*_ *i* ⋅ *ψ*_*mi*_ where *ψ*_*mi*_ is the normalized weight of the m^th^ eigenmode at position *i*.

We begin by developing the prescription of the topological pump on a 1D array of identical resonators having couplings with alternating strengths (dimerized) as depicted in Fig. 1a. Sub-lattices *A* and *B* correspond to the resonator positions inside a unit cell, with intra-cell coupling rate *γ* and inter-cell coupling rate *λ*. This system can be described through the well-known Su-Schreefer-Heeger model for polyacetylene^32,33^, which informs us of the existence of two distinct phases in the presence of inversion symmetry. The array is in a topologically non-trivial phase when *γ* < *λ*, protected by the approximate inversion or chiral symmetries, and is in a topologically trivial phase if *γ* > *λ*. A finite array composed of these unit cells in the non-trivial phase supports a mid-gap mode confined to each end of the chain. For a translationally invariant chain with periodic boundary conditions the Bloch Hamiltonian of this system is written as

1 $$H({k}_{x})=(\gamma +\lambda \cos ({k}_{x})){\sigma }_{1}+\lambda \sin ({k}_{x}){\sigma }_{2},$$

where *k*_*x*_ is momentum along the array, and $\sigma_{1}=({{0}\atop {1}}\; {{1}\atop {0}})$ and ${\sigma }_{2}=({{0}\atop {i}}\; {{-i}\atop {0}})$ are Pauli matrices. The above system can now be modulated to produce the dynamic equivalent of a 2D Chern insulator^12^ that is described by the momentum space Hamiltonian

2 $$H({k}_{x},\phi )=(\gamma +\lambda \cos ({k}_{x})+{\gamma }_{m}\cos (\phi )){\sigma }_{1}+\lambda \sin ({k}_{x}){\sigma }_{2}+\beta \sin (\phi ){\sigma }_{3}\,.$$

Here we have introduced *ϕ* as an effective momentum in a second, synthetic dimension. This parameter *ϕ* is in practice the angular phase of the pumping cycle (which varies from 0 to 2*π*) and is proportional to time. The modulation that introduces the Pauli matrix ${\sigma }_{3}=({{1}\atop {0}}\; {{0}\atop {-1}})$ corresponds to odd-symmetric frequency modulation of the sublattices. This term breaks inversion symmetry during the pumping cycle and ensures that the Hamiltonian remains gapped throughout. The parameters *γ*_*m*_ and *β* are the modulation depths of the coupling rates and the on-site potentials respectively.

Upon mapping from momentum space into real space, the Hamiltonian for the topologically pumped 1D array can be written as

3 $$H(\phi )= \, \sum _{n}\left((\lambda -{\gamma }_{m}\cos \phi ){a}_{n}^{\dagger }{b}_{n}+\lambda {b}_{n}^{\dagger }{a}_{n+1}+h.c.\right.\\ +\,\left.\beta \sin \phi ({a}_{n}^{\dagger }{a}_{n}-{b}_{n}^{\dagger }{b}_{n})+\frac{{\gamma }_{m}}{2}\cos \phi ({a}_{n}^{\dagger }{a}_{n}+{b}_{n}^{\dagger }{b}_{n})\right),$$

where *a*_*n*_(${a}_{n}^{\dagger }$) and *b*_*n*_(${b}_{n}^{\dagger }$) are the annihilation and creation operators of the modes of interest on the two sub-lattice sites within the *n*-th unit cell. We achieve the above prescription by keeping the inter-cell coupling *λ* fixed, while modulating the intra-cell coupling as $\gamma (\phi )=\lambda -{\gamma }_{m}\cos \phi$. Simultaneously, the on-site potentials are modulated as $\Delta {f}_{A}(\phi )=-\beta \sin \phi$ and $\Delta {f}_{\! B}(\phi )=+\beta \sin \phi$. The prescribed modulations of the coupling and on-site potentials are graphically illustrated in Fig. 1a. The last term in Eq. (3), which we did not include in Eq. (2), arises from behavior specific to our system^34^ as described in Supplementary Note 1. However, since this term is identical on all sites, it does not change the eigenmodes or any robust properties of the topological pump, but only acts to shift the mode frequencies as a function of the phase in the pumping cycle.

The pumping process can be illustrated as follows. Without loss of generality let the pumping phase *ϕ* = 0 or 2*π* represent the array in the topologically non-trivial phase, with two edge modes within the bandgap that are degenerate in frequency and positioned on opposite ends of the chain (see Supplementary Note 8). We identify these modes as the lower edge mode (EM_L_) and the upper edge mode (EM_U_), due to the paths in frequency they follow during the pumping cycle. As *ϕ* evolves away from 0, both EM_L_ and EM_U_ become dispersive and merge into the bulk with EM_L_ decreasing in frequency and EM_U_ increasing in frequency (Fig. 1b). At exactly mid cycle *ϕ* = *π* the array recovers inversion symmetry but is now in the topologically trivial phase. As *ϕ* continues evolving towards 2*π*, the edge modes re-emerge from the bulk bands, and have now migrated to the opposite physical ends of the array from where they started (Fig. 1c). Since *H*(*ϕ*) is gapped for all *ϕ*, we can calculate the Chern number of the pumping cycle, which for our system is 1 (see Supplementary Note 2). This means that the pumping process is topologically protected and EM_L_ and EM_U_ are topologically robust to disorder and smooth changes of system parameters.

### Experimental implementation

**Fig. 2:**
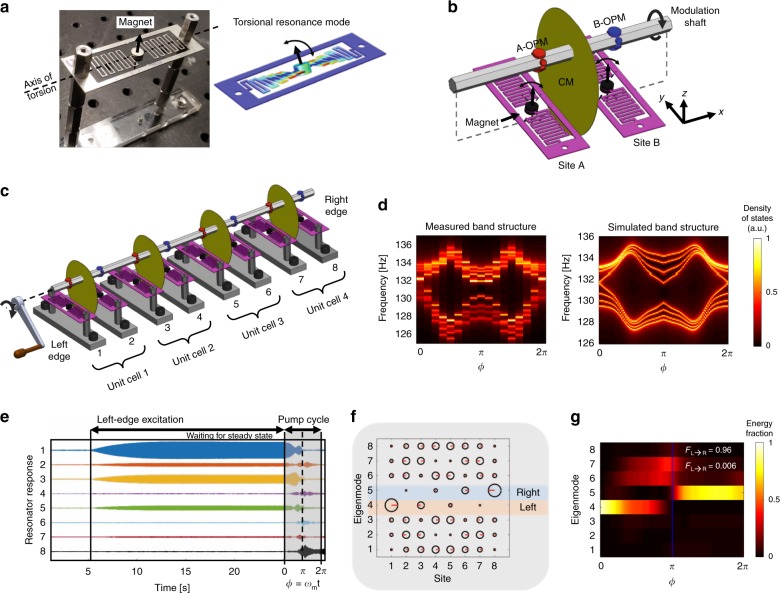
Magneto-mechanical system description. **a** Photograph of an individual magnet-loaded mechanical resonator and its simulated torsional resonance mode. **b** Each unit-cell comprises of two mechanical resonators (Sites A, B) that are coupled through magnetic interaction. Angular rotation *ϕ* of the modulation shaft (Supplementary Movie 1) simultaneously induces coupling modulation (CM) and on-site potential modulations (OPM). CM is generated with an off-axis high-permeability metal alloy sheet (Supplementary Note 7) while OPM utilizes permanent magnets (Supplementary Note 6). **c** All experiments use an array of 4 unit-cells. Rotation of the shaft is motorized, though in principle a crank handle (illustrated) could be used to activate the pump. **d** Band structure is measured quasi-statically by averaging the normalized mechanical impedance spectrum measured at each resonator, for discrete shaft angles *ϕ*. Experiments and simulations confirm edge states at *ϕ* = 0, 2*π* and an open bulk band gap for all *ϕ*. The simulation includes non-ideal effects such as next-nearest neighbor coupling (Supplementary Note 8). **e** In a typical pumping experiment, we simultaneously measure the deflection of all resonators. The array is driven only at site 1 to primarily excite the left edge mode. After stabilization, the drive is turned off and the pump (shaft rotation) is immediately activated. The example shows a pumping process with rotation rate *ω*_*m*_ ≈ 0.3 Hz. **f** Calculated eigenmodes of the eight resonator array at *ϕ* = 0. Circle size corresponds to the magnitude of the eigenmode while the red line indicates the corresponding phase (0 to the right, *π* to the left). The localized topological modes on the left and right edges (modes 4, 5) are highlighted. **g** Temporal heat map representing the modal energy fractions (*E*_mode *#*_) during the pumping process, obtained by projecting the measured motion in **e** on to the eigenmode basis in **f**. Mechanical energy is transported from the left edge mode (mode 4) to the right edge mode (mode 5) in one pumping cycle. Fidelity parameter *F*_*L*→*R*_ ≈ 1 (defined in text) indicates successful pumping.

We implemented the topological pump using an array of magnetically-coupled mechanical resonators. Each resonator (Fig. 2a) is identically fabricated from waterjet-cut aluminum. A neodymium magnet is bonded onto the central platform and serves both as the resonant mass as well as the mechanism by which adjacent resonators are magnetically coupled. The serpentine spring provides the restoring torque and sets the frequency for the torsional resonance mode at 132.4 Hz. The magnetically induced torque between the resonator dipoles couples their rotational degrees of freedom, and is used to produce the topological band structure. The coupling rate decays cubically with distance and can also be modified by placing high-permeability material between the resonators. Details on the magnetic interaction and the equations of motion specific to this system are presented in Supplementary Note 1. The typical −3 dB bandwidth of our resonators is Δ*f* ≈ 0.38 Hz which implies a decay time constant of *τ* = 1∕(*π*Δ*f*) ≈ 0.85 s. This timescale is not sufficient for an experimental observation of topological pumping since, as we discuss later (and in Supplementary Note 3) the adiabaticity timescale of the system is around 1.6 s. Therefore, for each resonator we implement an anti-damping circuit that provides a velocity-dependent feedback force to increase the decay time to 3.5 s (details in Supplementary Note 5).

A single unit cell of the array is comprised of two resonators as shown in Fig. 2b, corresponding to sub-lattice sites A and B. The experiment employed four unit cells as illustrated in Fig. 2c. Prior to experiments, we ensured the tuning of each resonator frequency to within +/−0.05 Hz measurement resolution. The anti-damping circuits were also individually tuned to provide a Q-factor of 1440 ± 100. This iterative process is very time consuming, e.g., can take several hours to complete for 8 resonators, and imposed a practical constraint on the experiment size (see also Supplementary Note 11). A photograph of the experimental setup is provided in the Supplementary Note 4. We physically implemented the pump cycle using a rotating shaft whose angular rotation directly represents the pump phase *ϕ* and which can be in essence ‘cranked’ whenever the pump needs to be activated. A clockwise (cw) rotation of the shaft corresponds to increasing *ϕ* from 0 to 2*π*, while counter-clockwise (ccw) rotation corresponds to decreasing *ϕ* from 2*π* to 0. This shaft is designed so that its rotation simultaneously produces the required coupling modulations and the required frequency (on-site potential) modulations without any physical contact with the resonator array, by means of only magnets and ferromagnetic materials. The on-site frequency modulations are implemented by leveraging the magneto-static spring effect^34^. This effect originates from the angular displacement-dependent torque acting on the magnetic harmonic oscillator in a non-uniform background magnetic field. Here we place permanent magnets on appropriate facets of the shaft to induce the required *ϕ*-dependent frequency modulation (see Supplementary Note 6 for details). We similarly modulate the intra-cell resonator coupling *γ* using high-permeability mumetal sheets mounted off-axis on the modulation shaft (Fig. 2b). During rotation these sheets enter the gap between the site A and B resonators and change the coupling as a function of *ϕ*. The specific geometry of the coupling modulation sheets is discussed in Supplementary Note 7. An animation of how the coupling-modulators and on-site potential modulators act during rotation of the shaft is provided in the Supplementary Movie 1. Each resonator is equipped with a Hall sensor that measures its angular displacement. All eight resonators in the array are measured simultaneously so that both the magnitude of displacement and the relative phase can be known. During experiments, the excitation of the mechanical motion of any resonator is achieved using a sinusoidal magnetic field produced by a drive coil placed nearby.

### Demonstration of topological pumping

We begin the experiment by performing a quasi-static characterization of the band structure of the magneto-mechanical states through the pumping cycle. The magneto-mechanical susceptibility (density of states) for any site in the array can be measured by actuating with a coil and measuring the calibrated angular displacement as a function of excitation frequency. These susceptibility measurements are then averaged over all resonators to produce a visualization of the mechanical density of states, as a function of shaft angular position, i.e., pump phase *ϕ*. The experimentally measured band structure for the system composed of 4 unit-cells is shown in Fig. 2d, and matches very well with the theoretical band structure which we modeled using couplings to nearest and next-nearest neighbors. This quasi-static measurement confirms that the band gap remains open throughout the pump cycle, and that mid-gap topological edge modes are present at *ϕ* = 0 and 2*π*. We provide additional discussion on this band structure in Supplementary Note 8.

We can now demonstrate the dynamic pumping cycle and show that the energy in the left edge mode is robustly transported across the array to the right edge. We start each pumping experiment by exciting the left edge resonator at the frequency of the topological edge mode. The excitation continues until a steady state response is reached. The excitation is then turned off and the modulation shaft is immediately activated to undergo one complete rotation, thereby evolving *ϕ* from 0 to 2*π*. An example of a typical measured angular displacement as a function of time for all 8 resonators is presented in Fig. 2e. In the representative example shown, mechanical energy is observed to transport across the array and localize on the right edge (resonator #8).

Of key interest to this study is to quantify the localization of the mechanical energy through the pumping cycle, with special attention placed on the two edge modes in the topologically non-trivial configuration at *ϕ* = 0 and *ϕ* = 2*π*. We therefore establish the *ϕ* = 0 eigenmode set (Fig. 2f) as a convenient basis in which we can analyze the modal energy distribution. Here we can also define an energy fraction for a mode (*E*_mode *#*_) as the fraction of total mechanical energy in the array projected onto the selected mode. The energy fraction for all 8 modes is traced throughout the pump cycle using overlapping 0.25 sec time segments (see Supplementary Note 9) – an example temporal heat map from an experimental measurement is presented in Fig. 2g. At the beginning of the pumping cycle the mechanical energy primarily sits on basis mode 4, corresponding to the left edge mode, while at the end of the cycle the energy transports to basis mode 5 which corresponds to the right edge mode. To further quantitatively analyze the pumping cycle we define a transport fidelity parameter *F*_*L*→*R*_ = *E*_5_(*ϕ* = 2*π*) ∕ *E*_4_(*ϕ* = 0) as the ratio between energy fraction in the right edge mode at the end of the cycle, and the energy fraction in the left edge mode at the beginning of the cycle. This parameter quantifies how much of the initial energy in the left edge mode has transported across the array, and is a measure of the performance of the pump. Similarly, the parameter *F*_*L*→*L*_ = *E*_4_(*ϕ* = 2*π*) ∕ *E*_4_(*ϕ* = 0) indicates how much mechanical energy remains in the left edge mode at the end of the cycle. In an ideal pump cycle we expect *F*_*L*→*R*_ = 1 and *F*_*L*→*L*_ = 0. For the specific example shown in Fig. 2g the measured transport fidelity is *F*_*L*→*R*_ = 0.96 demonstrating a successful pumping cycle. As we discuss below, the transport fidelity remains very high even in the presence of disorder as long as the adiabatic timescale is respected.

### Importance of adiabaticity

**Fig. 3:**
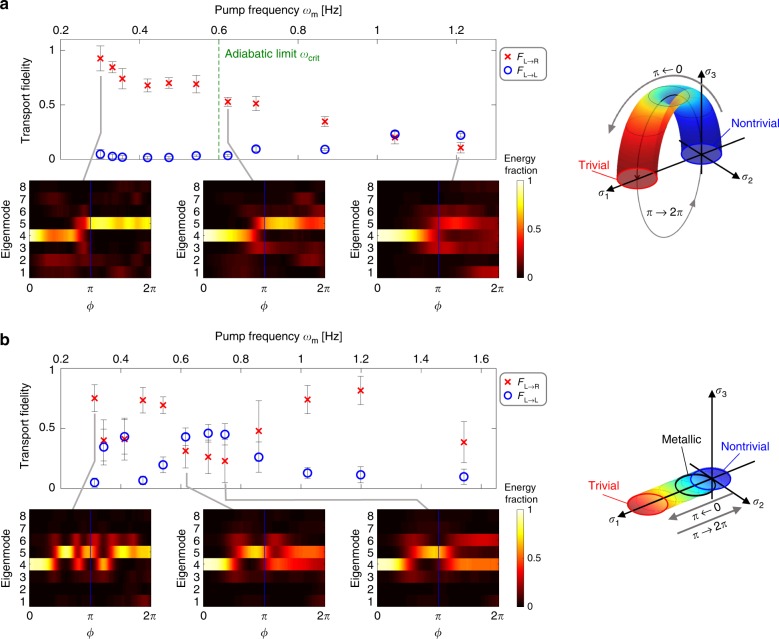
Experimental measurement of transport fidelity and exploration of the adiabatic regime. **a** We experimentally tested a range of pump frequencies *ω*_m_ traversing the critical threshold *ω*_crit_. For *ω*_m_ < *ω*_crit_ the transport fidelity *F*_*L*→*R*_ remains high, and diminishes for *ω*_m_ > *ω*_crit_. Each data point corresponds to the average of fidelity measurements from 10 consecutive pumping experiments and error bars show the standard deviation between experiments. The insets show representative temporal heat maps of the energy fraction of each basis mode in Fig. 2f. In the adiabatic regime, mechanical energy robustly transports from mode 4 (left edge) to mode 5 (right edge), while in the non-adiabatic regime the energy disperses amongst the bulk modes. The Pauli matrix basis representation of the pumping Hamiltonian is presented on the right, and shows how the Hamiltonian evolves during the pumping cycle between non-trivial and trivial phases while avoiding closing the band gap. **b** We also tested the non-adiabatic case where band gap closes twice during the cycle, as shown by the Pauli matrix basis representation on the right. In this case the Chern number cannot be used to characterize the process. Mechanical energy is seen to oscillate between the two edge modes, and therefore energy transfer is strongly dependent on timing. Supporting simulations for both cases are provided in Supplementary Note 10.

Having demonstrated on-demand temporal pumping in the magneto-mechanical resonator array, we turn to illustrate the importance of adiabaticity. The pumping process timescale is characterized by the frequency *ω*_m_ which also corresponds to the angular rotation rate *dϕ* ∕ *dt* of the shaft. Intuitively, the adiabatic condition is such that the frequency of the Hamiltonian modulations during the pumping process must be smaller than the frequency gap between a given eigenmode (EM_L_ or EM_U_ in our case) and the rest of the eigenmodes, to mitigate transitions between the modes. Quantitatively, we calculate a critical pump frequency *ω*_crit_ ≈ 0.6 Hz above which the adiabaticity of the system breaks down^35^ (calculation in Supplementary Note 3). We expect that it is only in the adiabatic regime that the pumping process is characterized by non-vanishing Chern number of 1 (Supplementary Note 2) and is therefore topologically protected. To show the breakdown of adiabaticity we experimentally measured values of *F*_*L*→*R*_ and *F*_*L*→*L*_ as a function of increasing pump frequency *ω*_m_. Figure 3a presents the measured fidelities averaged over 10 consecutive experiments. We observe that pumping is achieved (*F*_*L*→*R*_ approaches 1) below the theoretically calculated *ω*_crit_ ≈ 0.6 Hz, and diminishes past this threshold. The example insets show how the energy transports from mode 4 (left edge) to mode 5 (right edge) in the adiabatic pumping regime, but disperses amongst other bulk modes in the non-adiabatic pumping regime.

A limiting case where the adiabatic condition necessarily breaks is if the band gap closes at some point during the pumping cycle. In this situation, there is not a well-defined Chern number associated with the pumping process and the reliable transfer of energy between edge states requires precise timing since it is a result of the coupling between the two edge modes instead of a topological pump. To illustrate this non-adiabatic process, we modify the modulation shaft to turn off the resonator frequency modulations and only retain the coupling modulations. As a result, the Hamiltonian for the system (Eq. 2) no longer contains the *σ*_3_ term, and the band gap closes twice during the pump cycle, i.e., the system transits through a (bulk) conducting phase, as illustrated in Fig. 3b (see also Supplementary Fig. 9). Once again, Fig. 3b presents experimental measurements of the transport fidelity as a function of pump frequency *ω*_m_. The values of *F*_*L*→*R*_ and *F*_*R*→*L*_ are seen to be irregular with no clear regime of pump frequency separating high and low values. Moreover, the example insets show that the mechanical energy oscillates between the two edge modes (modes 4 and 5) during the cycle, confirming that the transport of mechanical energy is timing-dependent.

The results presented in Fig. 3 are for cw rotation of the modulation shaft (*ϕ* increasing), i.e., pumping along EM_L_. An additional set of experiments with ccw rotation (*ϕ* decreasing) implying a pumping trajectory along EM_U_ are presented in Supplementary Note 12 along with supporting simulations in Supplementary Note 10. As expected, ccw pumping also confirms the same adiabaticity characteristics.

### Robustness to spatio-temporal disorder

**Fig. 4:**
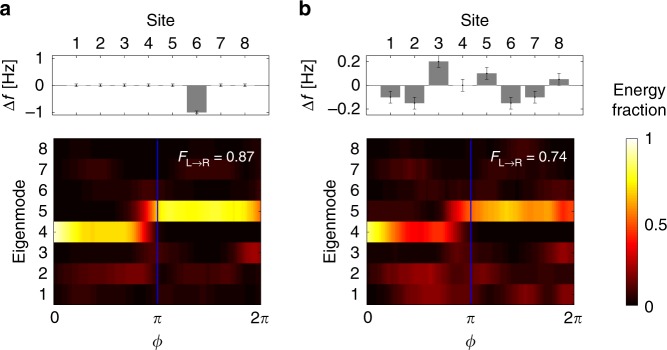
Pumping in the presence of static on-site potential disorder. **a** A single resonator is detuned by 1 Hz. **b** Resonators are randomly detuned by a range spanning  ±0.2 Hz from their common frequency, corresponding to  ~10% of the band gap. In both cases the temporal heat map of energy fractions shows robust pumping with high transport fidelity. The error bars show the 0.05 Hz frequency uncertainty in the on-site potential disorder due to the measurement limits.

Since the adiabatic pump is characterized by a non-vanishing Chern number, we expect the process to be robust to defects that deform the band structure but do not close the band gap. One class of static defects that satisfy this criterion is the detuning of on-site potential, for which we present two specific examples in Fig. 4. The first example has a single resonator frequency detuned by 1 Hz. The second example uses a randomized detuning of  ±0.2 Hz, which is quite large compared to a 2 Hz bandgap. Results from pumping experiments show high transport fidelity *F*_*L*→*R*_ for both cases. A wide range of additional experimental cases are presented in Supplementary Note 13 and exhibit consistent robustness against non-time-varying on-site potential disorder. Since the array size is limited in our experiments, we have added a discussion on larger disordered arrays in Supplementary Note 11.

**Fig. 5:**
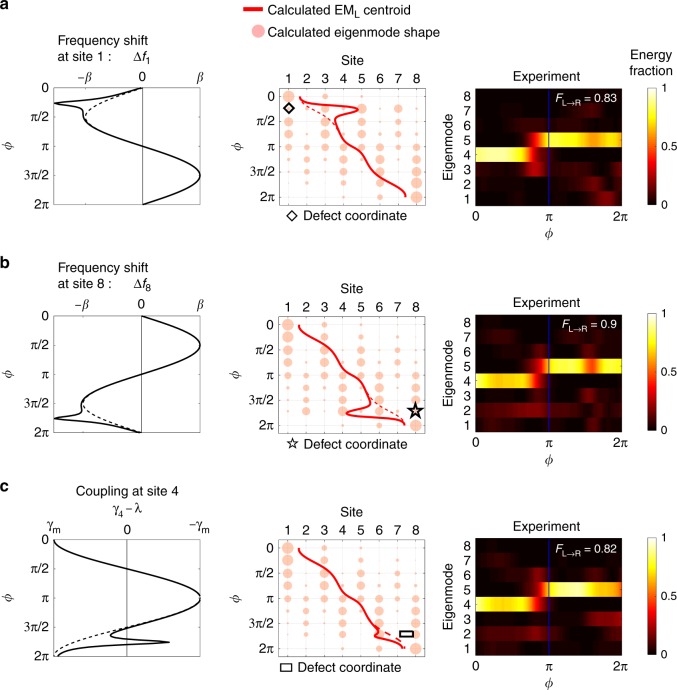
Implementation of spatio-temporal defects. Since the governing Hamiltonian of the topological pump matches that of a Chern insulator, pumping is robust to defects along its pseudo-edge in space and time. We can use the centroid of the EM_L_ mode to track this pseudo-edge and place defects along its path. **a** A temporal on-site potential defect is placed at the space (site) and time (*ϕ*) coordinates marked by the diamond symbol. The defect is produced by modifying frequency *f*_1_ away from the prescribed trajectory during the pumping cycle. The experimentally measured modal energy fraction exhibits high fidelity indicating that the pump remains robust. **b** We repeat the experiment from **a** with an on-site potential defect at the coordinates indicated by the star symbol. Again, high fidelity pumping is experimentally measured. **c** We also explored the intrusion of a trivial phase at the boundary of the equivalent 1 + 1D Chern insulator. This is implemented by modifying the intra-cell coupling rate *γ*_4_ at the 4th unit cell, between sites 7 and 8, with space-time coordinates marked by rectangle symbol. Here *γ*_4_ deviates from the prescribed trajectory during the pumping process (inter-cell coupling rate *λ* remains fixed). Once again, the experimentally measured modal energy fraction shows high fidelity pumping in spite of the trivial phase intrusion.

As mentioned previously, the Hamiltonian describing the system is effectively that of a Chern insulator with one real spatial dimension and one synthetic frequency dimension (See Eq. (3)). Therefore, the system should exhibit robustness against defects that deform the pseudo-space-frequency edge of the equivalent 1 + 1D Chern insulator. To find this pseudo-edge, we analyzed the spatio-temporal trajectory of the EM_U_ and EM_L_ modes by visualizing their centroids in space (resonator site) and time (pump phase *ϕ*) as shown in Fig. 1c for 100 sites and in Supplementary Fig. 14 for 8 sites. This visualization reveals the approximate space-time coordinates of mechanical energy through the pump cycle and helps to position the defects. At the beginning and the end of the cycle the mechanical energy is mostly localized near the left and right edge respectively, while in the middle of the cycle the mechanical energy propagates through the bulk. Based on this analysis, we experimentally implemented on-site potential defects to coincide with the EM_L_ centroid trajectory at site 1 at time *ϕ* = *π* ∕ 4, and at site 8 at time *ϕ* = 7*π* ∕ 4. The defects were designed to be a simple momentary perturbation of on-site potential (i.e., Δ*f*_1_, Δ*f*_8_ respectively in Fig. 5a, b) by modifying the permanent magnets on the shaft at the corresponding sites and phase angle *ϕ*. The experimental measurements presented in Fig. 5a, b show that pumping fidelity *F*_*L*→*R*_ remains very high in both of the above cases. Heuristically, the effective chiral edge mode simply avoids the defects and robustly pumps across the array without backscattering. A series of additional experiments implementing this type of temporal on-site potential defect are shown in Supplementary Note 13, Supplementary Figs. 16 and 17. Finally, we also implemented a coupling defect that mimics a phase boundary deformation, i.e. an intrusion of a trivial phase into the bulk of the 1 + 1D Chern insulator. This type of defect would act to deform the edge of the effective 2D system, and we would still expect the chiral edge state to adapt and travel around the new boundary geometry. This defect was implemented by momentarily increasing the intra-cell coupling *γ*_4_ at the 4th unit cell at time *ϕ* = 7*π* ∕ 4. An intuitive visualization of this defect is presented in Supplementary Fig. 18. Experimental results from pumping in this array (Fig. 5c) show that mechanical energy temporarily re-localizes in the penultimate unit-cell, but the overall transport fidelity *F*_*L*→*R*_ at the end of the cycle remains very high. All these experiments confirm the unique form of robustness of this topological pump against defects occurring in both space and time.

## Discussion

Linear waveguides are a foundational technology that enable modern systems for communications, sensing, and fundamental science. However, disorder that is frozen-in during fabrication, or appears dynamically in the form of fluctuations, can result in undesirable scattering^36–38^ and localization^39^ in these systems. While spatial topological pumps can address these concerns, the introduction of time as a pumping parameter offers unprecedented control and reconfigurability over the transport of energy in space^17,18^ and even in frequency^40^. Moreover, the use of alternative pumping protocols or multiple incommensurate temporal drives can potentially open up a wide configuration space^41,42^, allowing the synthesis of larger Chern numbers for increased pumping capacity^43–47^, the generation of higher Chern numbers in higher synthetic dimensions^48^, and the exploration of dynamic phase transitions between topological phases in time^49–51^.

## Supplementary information

Supplementary Information

Description of Additional Supplementary Files

Supplementary Movie 1

## Data Availability

The data that support the findings of this study are available from the corresponding author on reasonable request.
