# Supplementary material for: Robust temporal pumping in a magneto-mechanical topological insulator
Source: Nat Commun. 2020 Feb 20;11:974. doi: 10.1038/s41467-020-14804-0 (PMC7033202; doi:10.1038/s41467-020-14804-0)
Supplement: Supplementary file 1 — Supplementary Information [file 41467_2020_14804_MOESM1_ESM.pdf]

Supplementary Information: Robust temporal pumping in a magneto-mechanical topological insulator

Inbar Hotzen Grinberg[1], Mao Lin[2], Cameron Harris[2],
Wladimir A. Benalcazar[2], Christopher W. Peterson[3],
Taylor L. Hughes[2*], and Gaurav Bahl[1*]

[1] Department of Mechanical Science and Engineering, [2] Department of Physics,
[3] Department of Electrical and Computer Engineering,
University of Illinois at Urbana-Champaign, Urbana, Illinois 61801, USA
[*] To whom correspondence should be addressed; hughest@illinois.edu, bahl@illinois.edu

Supplementary Note 1

Equations of motion and derivation of system Hamiltonian

In this section we will derive the equations of motion of the magneto-mechanical resonator array, and reproduce the Hamiltonian presented in Equation 3 of the main text.

To model the magnetic interaction between the resonators, we consider each magnet-loaded resonator as a point dipole. This is heuristically acceptable as long as the distance between magnets is greater than their largest geometrical dimension. Generally, any magnetic dipole $\mathbf{m}$ placed within any magnetic field $\mathbf{B}$ feels a torque given by $\tau = \mathbf{m} \times \mathbf{B}$. Each point dipole is a source to a non-uniform magnetic field, and as a result the torque acting on dipole A due to dipole B is given by [1]

$$\tau_{\mathrm{AB}} = \frac{\mu_0}{4\pi r^5}(3\mathbf{m}_B \times (\mathbf{m}_A \cdot \mathbf{r})\mathbf{r} - r^2(\mathbf{m}_B \times \mathbf{m}_A)). \quad (1)$$

Here μ_0 is the magnetic permeability of free space, $\mathbf{m}_A, \mathbf{m}_B$ are the two magnetic dipoles, and $\mathbf{r}$ is their relative position (pointing from A to B).

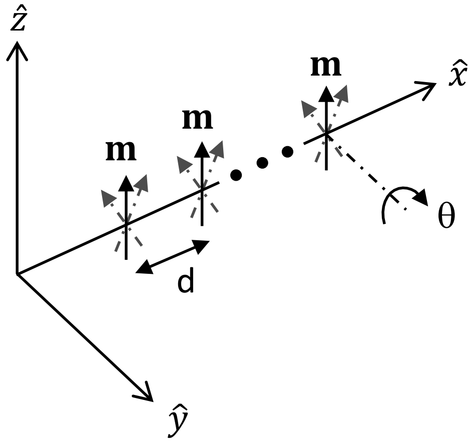

Supplementary Figure 1: Our system is composed of a linear array of magnetic resonators, each having a single rotational degree of freedom. This diagram accompanies the discussion in the text.

In our experimental setup all magnetic dipoles are oriented along the $\hat{z}$ axis at rest, i.e. $\mathbf{m} = m\hat{z}$, and are spaced only along the $\hat{x}$ axis, such that $\mathbf{r} = d\hat{x}$. Each dipole has a single rotational degree of freedom θ around the $\hat{y}$ axis, and we assume all dipoles are identical so that $m_A = m_B = m$ as illustrated in Supplementary Figure 1. We substitute these conditions into Supplementary Equation 1 and use a small angle approximation [2] to simplify the torque acting on dipole A due to dipole B to the following

$$\tau_{\mathrm{AB}} = \frac{2\mu_0 m^2}{4\pi d^3}\left(\frac{\theta_A}{2} + \theta_B\right). \quad (2)$$

Since the torque τ_{AB} on A is linearly dependent on the displacement θ_A, it corresponds to a linear spring term that acts in parallel with the mechanical spring κ of

that resonator. In addition, we note a second coupling term that applies a torque on A due to the angular displacement of B. The resulting coupled equations of motion for the A and B site resonators can then be written as:

$$\begin{cases} \ddot{\theta}_A + c\dot{\theta}_A + (\omega_0^2 - \frac{\gamma}{2})\theta_A - \gamma\theta_B = 0 \\ \ddot{\theta}_B + c\dot{\theta}_B + (\omega_0^2 - \frac{\gamma}{2})\theta_B - \gamma\theta_A = 0, \end{cases} \tag{3}$$

where

$$c = \frac{b}{I}\ ;\ \omega_0^2 = \frac{\kappa}{I}\ ;\ \gamma = \frac{2\mu_0 m^2}{4\pi d^3 I}. \tag{4}$$

Here b is the viscous damping coefficient, I is the rotational moment of inertia, and ω_0 is the natural resonance frequency of the mechanical resonator when isolated in space. The parameter γ is the magnetic interaction which induces both a magnetic spring effect and coupling between adjacent resonators [2]. The spring effect induced by this magnetic interaction results in the last term in Equation 3 in the main text. This effect is identical to all resonators in the array and therefore only induces a uniform frequency shift and will not result in closing of a band gap.

Now, given that A, B are the sublattice sites of a dimerized array with n unit cells having a fixed inter-cell coupling λ, a modulated intra-cell coupling $\gamma(\phi) = \lambda - \gamma_m \cos\phi$, and modulated resonance frequencies $\Delta f_{\mathrm{A,B}}(\phi) = \pm\beta\sin(\phi)$ we can write the equations of motion of the modulated array

$$\begin{cases} \ddot{\theta}_{n,A} + c\dot{\theta}_{n,A} + (\omega_r^2(\phi) - \beta\sin\phi)\theta_{n,A} - \lambda\theta_{n-1,B} - \gamma(\phi)\theta_{n,B} = 0 \\ \ddot{\theta}_{n,B} + c\dot{\theta}_{n,B} + (\omega_r^2(\phi) + \beta\sin\phi)\theta_{n,B} - \lambda\theta_{n+1,A} - \gamma(\phi)\theta_{n,A} = 0, \end{cases} \tag{5}$$

where the effective resonance frequency of each resonator in the array is

$$\omega_r^2(\phi) = \omega_0^2 - \lambda + \frac{\gamma_m}{2}\cos\phi. \tag{6}$$

Next, we invoke the slowly varying envelop approximation (SVEA) to reduce the order of the equations and write the system Hamiltonian. SVEA is the assumption that the envelope of the time domain amplitude changes slowly compared to the period of oscillations. Typical resonance frequencies of our resonators are around 130 Hz, while the modulation rate (which indicates the envelope) is ~ 1 Hz. This means there are two orders of magnitude difference between the timescale of the resonator oscillations and the time varying envelope, which justifies the use of SVEA. We now denote the oscillations of each resonator in the nth unit cell as $\theta_{n,\eta}$ with $\eta = A, B$, and assume that they take the following harmonic form

$$\theta_{n,\eta}(t) = x_{n,\eta}(t)e^{i\omega t} + c.c., \tag{7}$$

where ω is the frequency of the external drive, and $x_{n,\eta}(t)$ is the amplitude of oscillation. Taking the time derivatives of Supplementary Equation 7 we obtain

$$\begin{aligned} \dot{\theta}_{n,\eta}(t) &= \dot{x}_{n,\eta}(t)e^{i\omega t} + i\omega x_{n,\eta}(t)e^{i\omega t} + c.c. \\ \ddot{\theta}_{n,\eta}(t) &= \ddot{x}_{n,\eta}(t)e^{i\omega t} + 2i\omega\dot{x}_{n,\eta}(t)e^{i\omega t} - \omega^2 x_{n,\eta}(t)e^{i\omega t} + c.c.. \end{aligned} \tag{8}$$

Under SVEA we set $\ddot{x}_{n,\eta}(t) = 0$, and substitute Supplementary Equation 7-8 into Supplementary Equation 5. The equations of motion become

$$\begin{cases} 2i\omega\dot{x}_{n,A} - \omega^2 x_{n,A} + c(\dot{x}_{n,A} + i\omega x_{n,A}) + \left(\omega_r^2 - \beta\sin\phi\right) x_{n,A} - \lambda x_{n-1,B} - \gamma(\phi) x_{n,B} = 0 \\ 2i\omega\dot{x}_{n,B} - \omega^2 x_{n,B} + c(\dot{x}_{n,B} + i\omega x_{n,B}) + \left(\omega_r^2 + \beta\sin\phi\right) x_{n,B} - \lambda x_{n+1,A} - \gamma(\phi) x_{n,A} = 0. \end{cases} \tag{9}$$

Rearranging the equations yields

$$\begin{aligned} (i + \frac{c}{2\omega})\dot{x}_{n,A} &= \left(\frac{\omega^2 - \omega_r^2 + \beta\sin\phi}{2\omega} - \frac{ic}{2}\right) x_{n,A} + \frac{\lambda}{2\omega} x_{n-1,B} + \frac{\gamma(\phi)}{2\omega} x_{n,B} \\ (i + \frac{c}{2\omega})\dot{x}_{n,B} &= \left(\frac{\omega^2 - \omega_r^2 - \beta\sin\phi}{2\omega} - \frac{ic}{2}\right) x_{n,B} + \frac{\lambda}{2\omega} x_{n+1,A} + \frac{\gamma(\phi)}{2\omega} x_{n,A}. \end{aligned} \tag{10}$$

Since excitation frequency $\omega \approx \omega_0$, and losses are small $c \approx 0$, we obtain the dynamical equations

$$\begin{aligned} i\dot{x}_{n,A} &= \frac{1}{2\omega}\left(\left(\frac{\gamma_m\cos(\phi)}{2} + \beta\sin\phi\right) x_{n,A} + \lambda x_{n-1,B} + (\lambda - \gamma_m\cos(\phi) x_{n,B}\right) \\ i\dot{x}_{n,B} &= \frac{1}{2\omega}\left(\left(\frac{\gamma_m\cos(\phi)}{2} - \beta\sin\phi\right) x_{n,B} + \lambda x_{n+1,A} + (\lambda - \gamma_m\cos(\phi) x_{n,A}\right). \end{aligned} \tag{11}$$

This set of first order equations reveals all the couplings in the system and can be mapped to a Hamiltonian given by

$$\begin{aligned} H = \frac{1}{2\omega}\sum_n \Big(&(\lambda - \gamma_m\cos\phi) a_n^\dagger b_n + \lambda b_n^\dagger a_{n+1} + h.c. \\ &+ \beta\sin\phi(a_n^\dagger a_n - b_n^\dagger b_n) + \frac{\gamma_m}{2}\cos\phi(a_n^\dagger a_n + b_n^\dagger b_n)\Big), \end{aligned} \tag{12}$$

where $a_n(a_n^\dagger)$, $b_n(b_n^\dagger)$ are the standard annihilation and creation operators. Thus we recover Equation 3 of the main text.

Supplementary Note 2

Calculation of Chern number

For a one dimensional dimerized array subjected to periodic modulations, the most general Hamiltonian takes the form

$$H(k,\phi) = \epsilon(k,\phi) + \sum_{i=1}^{3} d_i(k,\phi)\sigma_i, \tag{13}$$

where the quasi-momentum and the angular position (k, ϕ) effectively define a two-dimensional parameter space. Here, σ_i represents the Pauli matrices $\sigma_1 = \left(\begin{smallmatrix} 0 & 1 \\ 1 & 0 \end{smallmatrix}\right)$, $\sigma_2 = \left(\begin{smallmatrix} 0 & -i \\ i & 0 \end{smallmatrix}\right)$, $\sigma_3 = \left(\begin{smallmatrix} 1 & 0 \\ 0 & -1 \end{smallmatrix}\right)$, and $d_i(k,\phi)$ are the components of a vector $\hat{d}$ that describes the Hamiltonian in the Pauli matrix space. As we are following a state

adiabatically to complete the pumping process, $\epsilon(k,\phi)$ only contributes to the dynamical phase [3], and does not affect the Chern number. This is also demonstrated by the Chern number definition

$$\nu = \frac{1}{4\pi}\int dk\, d\phi\, \hat{d}\cdot\frac{\partial\hat{d}}{\partial k}\times\frac{\partial\hat{d}}{\partial\phi}, \tag{14}$$

which is independent of $\epsilon(k,\phi)$. Writing the Hamiltonian for our system in momentum space, we find that the vector $\hat{d}$ is given by

$$\hat{d} = \{\lambda + \lambda\cos(k) - \gamma_m\cos(\phi), \lambda\sin(k), \beta\sin(\phi)\}\,. \tag{15}$$

In the Pauli matrix space this vector is represented by a torus enclosing the origin as illustrated in Supplementary Figure 2. Due to the σ_3 component of the vector, the path of the Hamiltonian never crosses through the origin which is the singularity where the band gap closes.

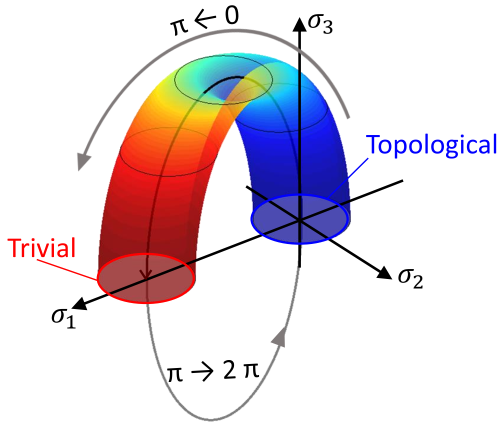

Supplementary Figure 2: A visual representation of the vector $\hat{d}$ which describes the modulated Hamiltonian of the topological pump (Supplementary Equation 15) in the Pauli matrix space.

Following the definition given in Supplementary Equation 14 the Chern number is analytically calculated for our system as

$$\nu = \begin{cases} -1, & \lambda < -\frac{\gamma_m}{2} \\ 0, & -\frac{\gamma_m}{2} < \lambda < \frac{\gamma_m}{2} \\ 1, & \lambda > \frac{\gamma_m}{2} \end{cases} \tag{16}$$

In our experimental system the values of the parameters are in the range of $\lambda > \frac{\gamma_m}{2}$ and therefore the Chern number is 1.

For the non-adiabatic process demonstrated in the main text the σ_3 term is zero since there is no on-site potential modulation, and the vector $\hat{d}$ becomes

$$\mathbf{d} = \{\lambda + \lambda\cos(k) - \gamma_m\cos(\phi), \lambda\sin(k), 0\}\,. \tag{17}$$

This suggests the band structure is gapless, and confines the system to lie in the

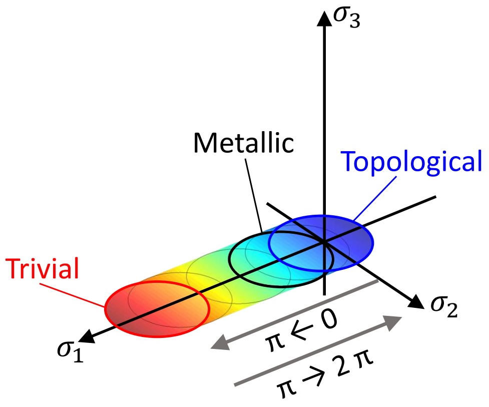

Supplementary Figure 3: A visual representation of the vector $\hat{d}$ which describes the modulated Hamiltonian of the non-adiabatic process (Supplementary Equation 17) in the Pauli matrix space (with no on-site potential modulations).

horizontal $\sigma_1 - \sigma_2$ plane as shown in Supplementary Figure 3. Calculating the Chern number for this process yields $\nu = 0$. Fundamentally, this is the result of symmetry. Since in this non-adiabatic process the mirror symmetry is preserved, and has the following representation

$$M_x = \sigma_1, \tag{18}$$

such that

$$M_x : H(k, \phi) \to H(-k, \phi) \tag{19}$$

throughout the process. Consequently we have $d_i(k, \phi) \to d_i(-k, \phi)$ under the reflection symmetry. Calculating the Chern number (using Supplementary Equation 14) for this system, we find that it flips sign under reflection symmetry, and thus is always 0 if reflection symmetry is preserved. The transport of energy in this case may still occur due to Rabi-like oscillations between the two coupled edge modes when the band gap shrinks or closes. This transport is therefore heavily dependent on timing as shown by the experimental results presented in Figure 3 (main text), and Supplementary Figure 13.

Supplementary Note 3

Discussion on adiabaticity

The adiabatic theorem, as it was originally proposed, states that a physical system remains in its instantaneous eigenstate if a given perturbation is acting on it slowly

enough and if there is a gap between the eigenvalue and the rest of the Hamiltonian's spectrum [4]. In this section, we shall review the derivation of the adiabatic theorem and analytically establish the adiabatic limit for our dimerized array. We shall show that the calculated critical pump frequency, beyond which adiabaticity fails, agrees with our experimental result quantitatively.

We start by considering a general time-varying Hamiltonian $H(t)$, and its instantaneous eigenstates

$$H(t)\psi_n(t) = \epsilon_n(t)\psi_n(t), \tag{20}$$

where the subscript n indicates an eigenstate. For a generic process starting with $\psi_n(t=0)$, after some time, typically the final state will not be $\psi_n(t)$ but rather a linear combination of all eigenstates. Therefore, for a process to be adiabatic, i.e. the nth eigenstate remains as the instantaneous state, it should have negligible probability to transition to any other state $\psi_m(t)$ in the spectrum. This means that the *change* of $\psi_n(t)$ should have negligible overlap with any other state. A mathematical description of this condition is therefore

$$||\langle\psi_m(t)|\partial_t\psi_n(t)\rangle|| \ll 1, \tag{21}$$

for all $m \neq n$ and at each time instance t, where $||\cdot||$ denotes the absolute value. Equivalently, by writing $|\psi_n(t+\delta t)\rangle = |\psi_n(t)\rangle + \delta t|\partial_t\psi_n(t)\rangle$, we get the following condition

$$||\langle\psi_m(t)|\psi_n(t+\delta t)\rangle|| \ll \delta t, \tag{22}$$

where we used the orthogonality of the eigenstates $\langle\psi_m(t)|\psi_n(t)\rangle = 0$. Supplementary Equation 22 states that the overlap between $|\psi_m(t)\rangle$ and $|\psi_n(t+\delta t)\rangle$, which is at the next instance in time, should be much smaller than the time-step. In fact, such overlap is closely related to the energy gap between the two states. To see that, we substitute Supplementary Equation 20 and rewrite Supplementary Equation 22 as

$$\left|\left|\frac{\langle\psi_m(t)|H(t) - H(t+\delta t)|\psi_n(t+\delta t)\rangle}{\epsilon_m(t) - \epsilon_n(t+\delta t)}\right|\right| \ll \delta t, \tag{23}$$

which we then rearrange as

$$\left|\left|\langle\psi_m(t)|\frac{H(t) - H(t+\delta t)}{\delta t}|\psi_n(t+\delta t)\rangle\right|\right| \ll ||\epsilon_m(t) - \epsilon_n(t+\delta t)|| \,. \tag{24}$$

By taking the limit $\delta t \to 0$ results in

$$\left|\left|\langle\psi_m(t)|\dot{H}(t)|\psi_n(t)\rangle\right|\right| \ll ||\epsilon_m(t) - \epsilon_n(t)|| \,, \tag{25}$$

which recovers the standard adiabatic condition [3]. Intuitively, $\dot{H}(t)$ serves as the *perturbation* that allows the transition between two instantaneously orthogonal states $\psi_{m,n}(t)$, which is otherwise forbidden for time-independent Hamiltonians. The

value of this perturbation is closely related to the pump frequency ω_m. This can be seen by taking the time derivative of the Hamiltonian given in Supplementary Equation 12, where the position in the pumping cycle is given by $\phi = \omega_m t$. We can follow the state $\psi_n(t)$ adiabatically, given that the perturbation or the rate of change of the Hamiltonian is smaller than the energy gap between $\psi_n(t)$ and *any* other states.

We note that Supplementary Equation 25 must hold for all $m \neq n$ at all times t. On the other hand, if there is a state that is degenerate with $\psi_n(t)$ at some time t^*, then the adiabatic theorem will break for $t = t^*$. This corresponds to the non-adiabatic pumping process discussed in the main text. In that experiment we turned off the frequency modulations and kept only the coupling modulations. During the pumping cycle when ϕ evolves from 0 to 2π the band gap closes twice, as shown in Supplementary Figure 9 and Supplementary Figure 3. As a result the whole process is non-adiabatic.

We use parameters based on our topological pumping experiment and plot the condition in Supplementary Equation 25 for different values of ω_m in Supplementary Figure 4. In the blue regions the value of perturbation is smaller than the energy gap and the condition in Supplementary Equation 25 holds, while in yellow regions it does not. The lowest value of the pump frequency for which adiabaticity breaks ($\omega_{\text{crit}} \approx 0.6$ Hz), sets the critical limit for the process, separating between adiabatic and non-adiabatic regimes. This analytical result agrees very well with the experiments in the main text (Figure 3) and in Supplementary Figure 13, where the normalized fractional energy $F_{L\to R}$ drops significantly around $\omega_m \approx 0.6$ Hz.

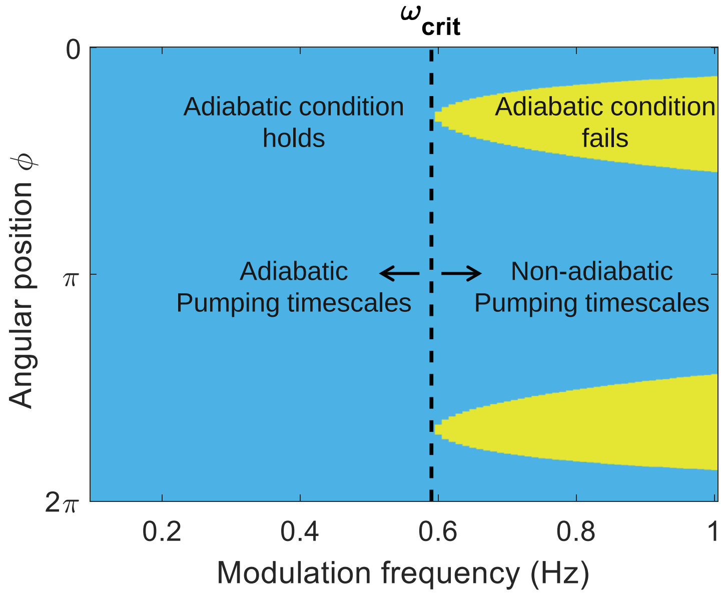

Supplementary Figure 4: Simulated adiabatic condition given by $||\langle\psi_m(t)|\dot{H}(t)|\psi_n(t)\rangle|| - ||\epsilon_m(t) - \epsilon_n(t)||$ for parameters matching our experimental system. The blue region is where the difference is negative and the adiabatic condition holds, and the yellow region is where it does not. The dashed black line marks the critical pump frequency $\omega_{\text{crit}} \approx 0.6$ Hz, beyond which the the adiabatic condition fails for some phases ϕ.

Supplementary Note 4

Experimental setup

The experimental setup composed of an 8 resonator array is shown in Supplementary Figure 5. The modulation shaft is positioned above the resonators and is connected to a motor at one end and a bearing at the other end to allow free rotation. Four mumetal sheets are connected to the shaft and positioned between A and B sites in each unit-cell to function as the coupling modulators. A series of permanent neodymium magnets (N52 material) are glued to the shaft at the resonator positions and induce the required on-site frequency modulations. Each resonator is equipped with a Hall effect sensor (not shown) to measure the change in magnetic field and infer angular displacement.

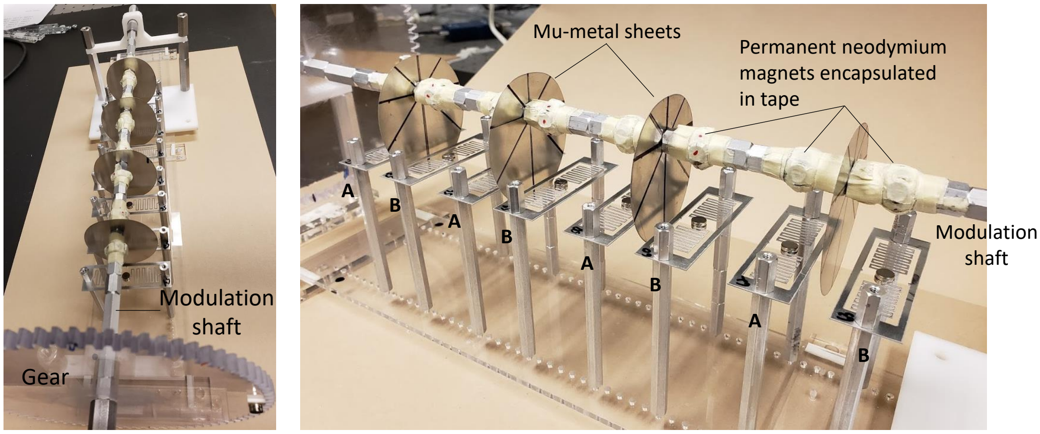

Supplementary Figure 5: Photograph of the 8 resonator array experimental setup.

Supplementary Note 5

Resonator characterization

The typical -3 dB bandwidth of our mechanical resonators is $\Delta f \approx 0.38$ Hz, which results in a decay time of the mechanical vibrational energy of $\tau = 1/(\pi \Delta f) \approx 0.85$ sec. The adiabatic limit calculated for our system in Supplementary Note 3 is 0.6 Hz, implying that the pumping cycle time must be 1.7 sec or longer. This means that the resonator decay timescale is not sufficient for convenient experimental observation of the pumping process.

In order to increase the decay time of the resonators, we implemented a feedback anti-damping circuit for each resonator (Supplementary Figure 6a). The output voltage from the Hall effect sensor of each resonator (equivalent to angular displacement θ) is fed back after amplification and a $\pi/2$ phase shift (equivalent to angular velocity $\dot{\theta}$) to a compact solenoid coil adjacent to the resonator. Since this force

feedback is proportional to $\dot{\theta}$, it can reduce the action of the viscous damping c (in Supplementary Equation 3) and increase the effective Q-factor and decay time of the resonator. The remainder of the resonator dynamics e.g. frequency, remain unchanged.

Typical results of a resonator without feedback are compared to three different feedback settings in Supplementary Figure 6c. The corresponding values of -3 dB bandwidth and decay times are reported in the table in Supplementary Figure 6b. In the experiments presented in the main text we choose to apply the feedback setting of level 2 to the resonators to set a decay time of 3.5 sec. We choose this level for our experiments since it provides sufficient decay time to perform pumping in the adiabatic regime while avoiding undesired complications. These complications include ambient noise, which can be amplified by the feedback circuit and can drive undesired modes, and the possibility of self-oscillation, which can occur when the feedback gain overcompensates for the intrinsic loss.

(a)

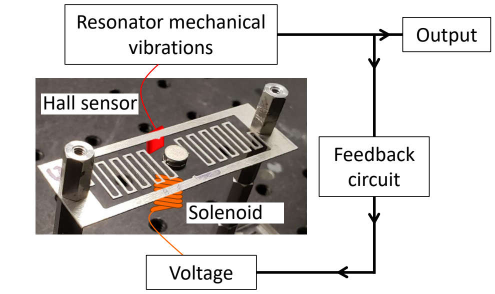

(b)

	-3dB bandwidth Δf [Hz]	Decay time [Sec]
No feedback	0.38	0.85
Gain level 1	0.13	2.2
Gain level 2	0.09	3.5
Gain level 3	0.07	4.1

(c) (d)

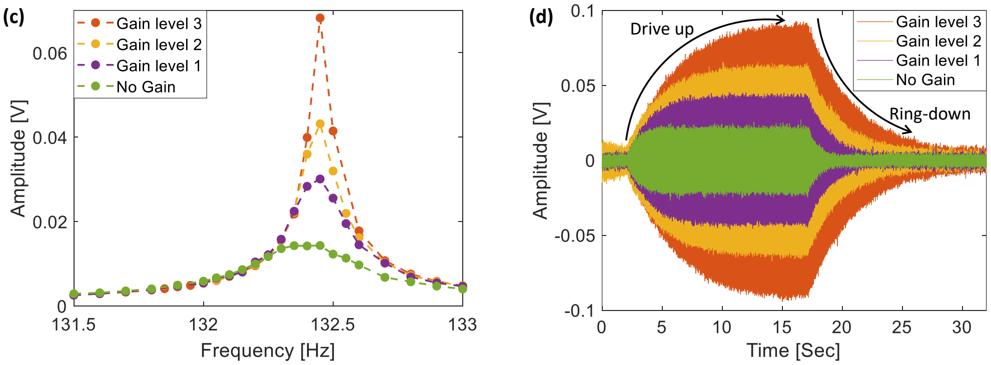

Supplementary Figure 6: (a) Schematic of the anti-damping feedback system. (b) Table summarizing the -3 dB bandwidth and decay times for a resonator without feedback and with three different feedback settings. (c) The frequency response of a resonator without feedback and with the three different feedback settings and (d) ring down measurements when excited at the resonance frequency.

Supplementary Note 6

Frequency modulation

We achieve frequency modulations in the resonator array through the magnetostatic spring effect (see Supplementary Note 1). A sequence of permanent magnets are attached to the circumference of the shaft above each resonator to function as the on-site potential modulator (OPM). As the shaft rotates, different magnets come into proximity with the resonator and induce an on-site frequency shift. The A and B sites are modulated with magnets of the same magnitude but opposite phasing as shown in Supplementary Figure 7a. Measured values of resonance frequency of site A as a function of the angular position ϕ are presented in Supplementary Figure 7b.

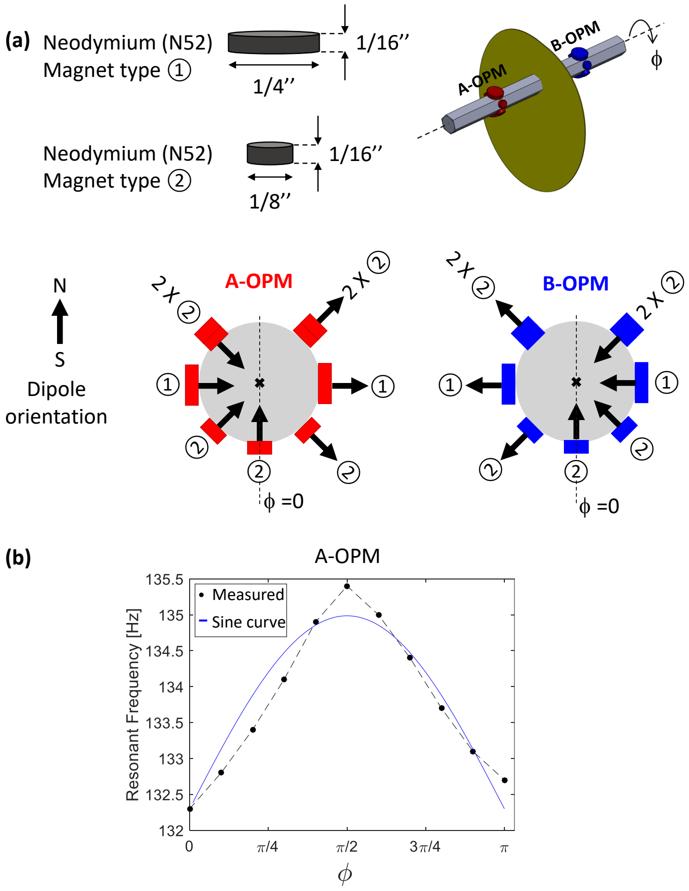

Supplementary Figure 7: (a) Illustrations of the magnet arrangement on the modulation shaft circumference used for on-site potential (frequency) modulations. Two different sizes of magnet were used and their arrangement for site A (A-OPM) and B (B-OPM) is with opposite phase. (b) Measured values of the resonance frequency of site A as a function of the modulation angle ϕ.

Supplementary Note 7

Coupling modulation

In order to modulate the coupling between resonators in a unit cell we use mu-metal (ferromagnetic material with high permeability) sheets, that divert the magnetic field between the resonators and reduce the magnetic coupling. The shape of this coupling modulator (Supplementary Figure 8a) was determined through experimental iterations. To measure the coupling rates experimentally we use a two resonator setup and evaluate the mode splitting. The measured coupling rates as a function of the angular position of the mu-metal sheet ϕ are shown in Supplementary Figure 8b.

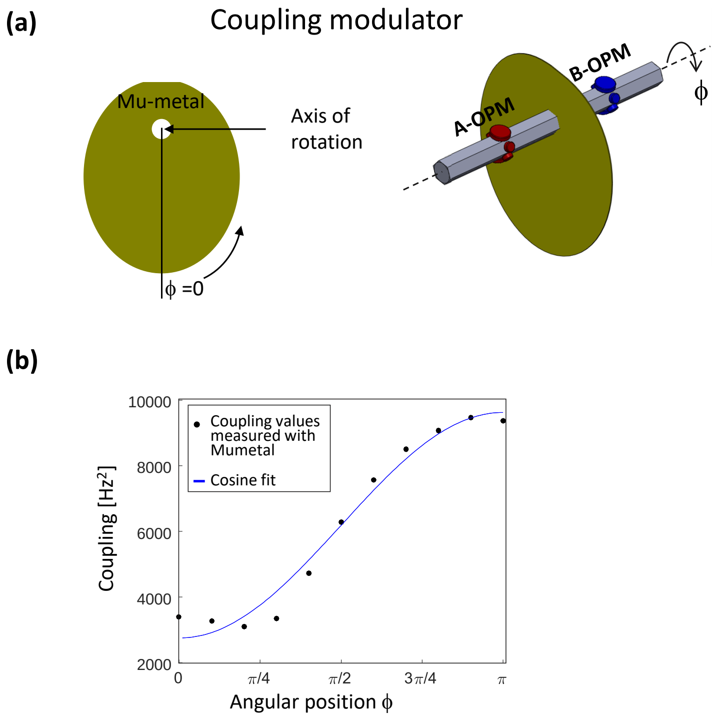

Supplementary Figure 8: (a) Design of the mu-metal coupling modulator to achieve the desired cosine modulation. (b) Experimentally measured coupling rate between resonators as function of the angular position of the modulation shaft.

Supplementary Note 8

Band structure

In this section we will present simulated and measured band-structures of our system, for both the topological pumping and for the non-adiabatic process described

in the main text and Supplementary Note 2. We will discuss some effects that influence our experimental system that are not included in the ideal model used to calculate the band structure presented in Figure 1b in the main text.

The topological pump is described by the Hamiltonian in Supplementary Equation 12, where resonance frequencies (on-site potentials) as well as the coupling rates are being modulated. In this case the band-gap does not close during the pumping cycle. As long as the adiabatic condition (Supplementary Note 3) is satisfied, it is guaranteed that we follow the same eigenstate from one edge of the array to the other, and transport the vibrational energy. A non-adiabatic process is demonstrated by a system where only the coupling rates are modulated. In this process the band gap closes twice in a cycle and therefore the adiabatic window collapses. In such a process, energy may oscillate between the two degenerate edge modes in a manner similar to Rabi oscillations. The two different mechanisms are explained in the main text and illustrated in Figure 3 (main text) as well as in Supplementary Figure 2 and Supplementary Figure 3 in Supplementary Note 2.

In Supplementary Figure 9 we present plots of the eigenfrequencies of the 1D array throughout the pumping process. We first simulate the eigenvalues for a 100 site (50 unit cells) lossless system as a function of the pumping parameter ϕ (Supplementary Figure 9a). At $\phi = 0, 2\pi$ the system is in the topologically nontrivial phase with two degenerate edge modes within the bulk band gap. In the topological pump the degeneracy of these edge modes is lifted for $\phi > 0$ due to the frequency modulations which break inversion symmetry, and the bandgap remains open throughout the pump cycle. In the non-adiabatic process the two edge modes stay degenerate until the bandgap closes, which happens twice during the pumping cycle.

Next, we wish to simulate approximately the band structure of our experimental system. We begin by simulating an array of 8 sites, and include a loss parameter evaluated based on experimental measurements (Supplementary Figure 9b). For a system which includes loss we can no longer calculate real eigenvalues. We therefore simulate the mechanical density of states (equivalent to mechanical susceptibility defined as the torque-to-angular-displacement transfer function) of the array at each value of ϕ. Repeating this process for values of ϕ in the range of $[0, 2\pi]$ visualizes the band structure of the system.

In this simulation we observe a similar trend as the case with 100 sites. We now also include in the simulations a few additional effects that are inevitable in any experiment, as shown in Supplementary Figure 9c. The first is next nearest neighbor coupling which we estimate based on measured values and the cubic decay of the magnetic coupling with distance. A second effect is that the modulations do not follow a perfect sinusoidal curves. We experimentally extract modulation functions based on fits to the measured values (see Supplementary Note 6 and Supplementary Note 7). These effects change the band structure slightly and better approximate the actual experimental measurements which are shown in Supplementary Figure 9d.

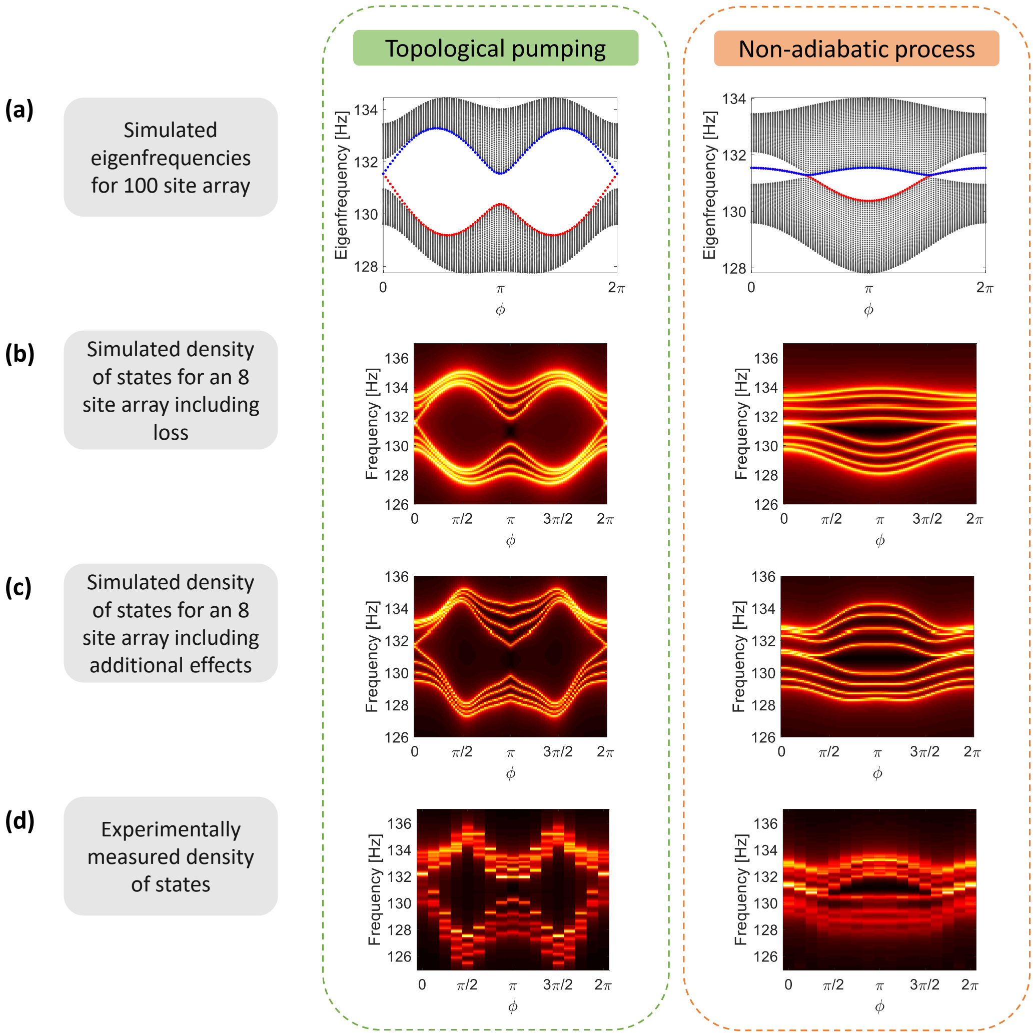

Supplementary Figure 9: Simulated and measured eigenfrequencies of the 1D modulated array for both the topological pump (left) and the non-adiabatic process (right). (a) Simulated eigenfrequencies as a function of the pumping parameter ϕ for an ideal array of 100 sites. The lower ($\mathrm{EM_L}$) and upper ($\mathrm{EM_U}$) paths of the edge modes are colored red and blue respectively. (b) Simulated eigenfrequencies of an 8 site array including intrinsic resonator loss. Brighter color represents greater density of states. (c) Simulated eigenfrequencies of an 8 site array including loss, next-nearest neighbor coupling, and frequency and coupling modulations based on experimental calibration. (d) Experimentally measured band-structure as a function of the pump angular position ϕ. Due to the symmetry of the system the measurements were taken in the range $\phi = [0, \pi]$ and mirrored for the plot.

The experimentally measured spectrum of all 8 resonator sites at $\phi = 0$ is presented in Supplementary Figure 10. Resonators 1 and 8 on the edges of the array show a prominent mode localized within the bulk band gap. The spatial distribution of the integrated energy (angular oscillation amplitude squared) is shown on the right panel of Supplementary Figure 10 where circle size corresponds to magnitude. While for the lower and upper bands the distribution is almost uniform throughout

the array, for the mid gap frequency range the energy is strongly localized at the two edges.

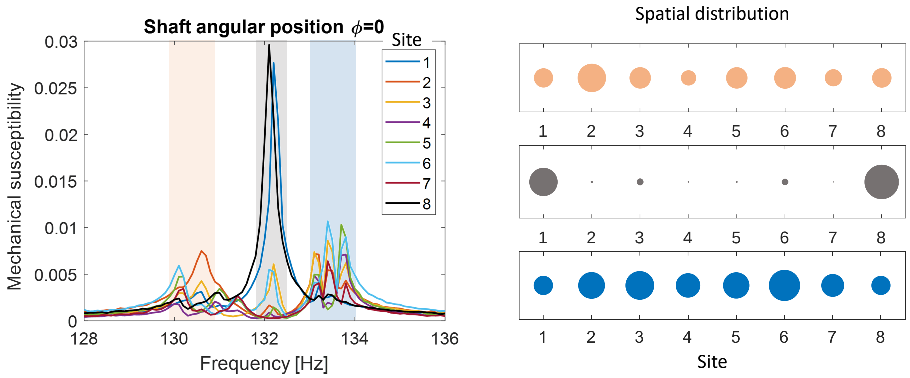

Supplementary Figure 10: Characterization of the array at $\phi = 0$. The experimentally measured spectrum of all 8 resonators is presented on the left, showing the mechanical susceptibility (ratio of angular displacement amplitude to applied torque, in arbitrary units) as a function of the drive frequency at each resonator. These measurements were performed by locally exciting each resonator with a small solenoid coil and measuring its mechanical response. The spatial distribution of the energy over the three frequency ranges highlighted on the spectrum plot is shown on the right. Circle size corresponds to energy magnitude averaged over the highlighted frequency range for each site.

Supplementary Note 9

Eigenmode decomposition

A typical measurement from our pumping experiment as presented in Figure 2e of the main text includes the vibrational motion of each resonator in the array as a function of time. The harmonic displacement $x_n(t)$ of the nth resonator can be written as the superposition of the system's eigenmodes such that $x_n(t) = \sum_{m=1}^{N} \psi_{\text{mn}} a_m(t)$. Here ψ_{mn} is the shape contribution of the mth eigenmode at the nth resonator (i.e. components of the eigenvectors), and a_m is its amplitude. By taking the Fourier transform of this equation we find the frequency domain expression $X_n(\omega) = \sum_{m=1}^{N} \psi_{\text{mn}} A_m(\omega)$. We can write this relationship in the matrix form $X = \Psi A$ where X is a column vector $[X_1(w), X_2(w)...]^T$ and A is a column vector $[A_1(w), A_2(w)...]^T$. We can then extract the eigenmode spectra from the measured displacement spectra using the inverse relation $A = \Psi^{-1} X$. The square of the spectrum A^2 is equivalent to the vibrational energy in each eigenmode. We define an energy fraction for each eigenmode ($E_{\text{mode }\#}$) as the fraction of total mechanical energy in the array projected onto the selected mode. We repeat this computational process for overlapping time segments of 0.25 sec throughout the pumping cycle, and track the energy in the different eigenmodes throughout the process. A typical result of this analysis is shown in Figure 2g of the main text.

Supplementary Note 10

Simulations of the transport fidelity

In this subsection we present simulation results of the transport fidelity values $F_{L\to R}, F_{L\to L}$. We simulate both the topological pump in which both frequencies and coupling values are modulated as well as the limit case of a non-adiabatic process where only coupling values are modulated, as discussed in the main text. To produce these simulations we first use a time domain solver for the full nonlinear equations of motion of the 8 resonator array. The resulting vibrational motion of all resonators is obtained, similar to the data we obtain experimentally (Figure 2e in the main text). We then repeat the eigenmode decomposition process explained in Supplementary Note 9, and calculate the transport fidelity values as defined in the main text. This simulation is repeated for many different pump frequencies, and the results are presented in Supplementary Figure 11. For topological pumping the energy is reliably transported from the left edge to the right edge of the array for a range of pump frequencies up to a critical value ω_{crit}. In contrast, for the non-adiabatic process, energy oscillates between the two edges due to Rabi-like oscillations. At the end of the non-adiabatic cycle the energy can be localized at either edge and is heavily dependent on timing, as shown by the oscillating values of $F_{L\to R}, F_{L\to L}$.

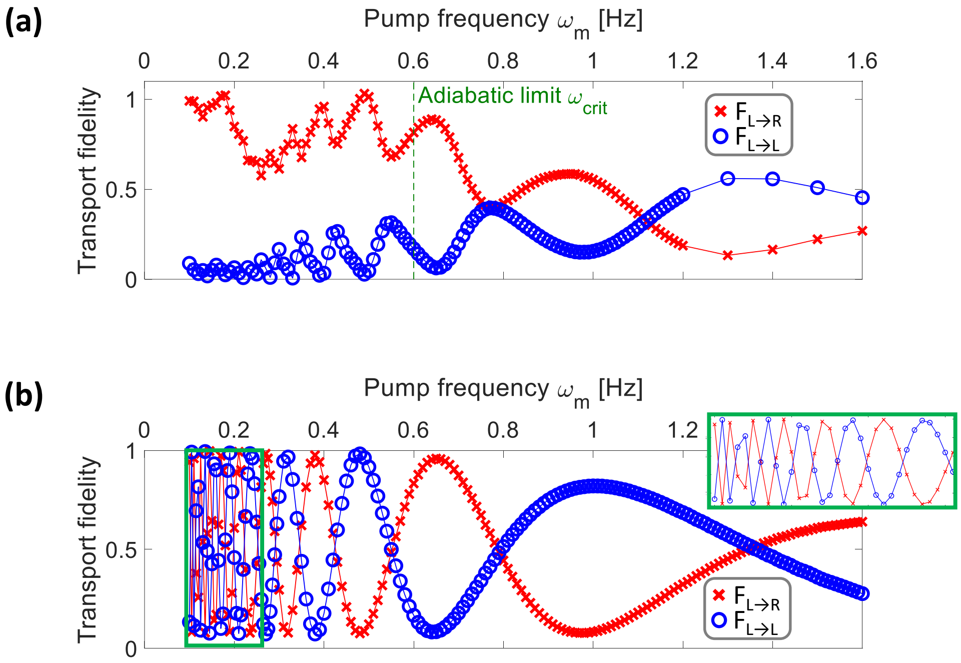

Supplementary Figure 11: Transport fidelity derived from simulations of an 8 resonator system as a function of the pump frequency ω_m. (a) Simulation of a topological pump and (b) of a non-adiabatic process.

Supplementary Note 11

Discussion on disorder and simulation of longer arrays

Experimental array size limitation – The resonators composing our system are made from 0.4 mm thick aluminum which is cut using a waterjet process. A neodymium magnet is then manually glued onto the central platform. Due to fabrication tolerances and manual handling of the resonators their natural frequencies exhibit some variation. Moreover, these frequencies can sometimes vary over time and due to environmental conditions. As a result, each resonator in the array requires an extensive individual tuning calibration prior to the experiment. The frequency tuning is performed individually to each resonator when it is isolated from any other parts of the system. Equally important is matching the Q-factors of the resonators, for which we manually adjust the feedback gain on each anti-damping circuit (see Supplementary Note 5) dedicated to each individual resonator. Every time that we wish to measure frequency and Q-factor after such adjustment, we must perform a frequency sweep analysis which takes several minutes to complete. The calibration and tuning process can take almost an entire day of experimental time even with only 8 resonators present.

Minimum amount of disorder in the experiments – Due to measurement resolution limits, the natural frequency of each resonator is only readily determined to within $\pm$ 0.05 Hz. This error bar has a total size of 0.1 Hz, which corresponds to 5% of the system band gap ($\sim$2 Hz) even without the introduction of any intentional disorder. An additional source of disorder is the cubic decay of the coupling rate which implies that the system is sensitive to any disorder in the resonator-resonator distances. Finally, the coupling modulators and frequency modulators are assembled by hand and are therefore not all identical, leading to further disorder in frequencies and coupling rates, which are not possible to experimentally determine. The results shown in Supplementary Figure 2g (main text) are therefore at the minimum level of disorder that can be experimentally tested.

Simulations of larger disordered arrays – Due to the experimental constraints on array length, we performed a few simulations to discuss how disorder affects longer disordered arrays. In Supplementary Figure 12 we present a series of simulated bandstructures for increasingly large resonator arrays subject to the pumping cycle, and with increasing levels of disorder. This frequency disorder is added using a uniform random distribution over the $\pm$ ranges shown in the column headers of Supplementary Figure 12. In each column, we present representative examples of the resulting pump cycles. We argue that, for even reasonably large amount of disorder, the pumping cycle can always be accomplished as long as we find an appropriate pumping frequency that satisfies the adiabatic condition. A more subtle qualitative observation is that as the system size is increased, the bandstructure appears to become more stable for the same level of disorder. More importantly, as the array size gets longer, the boundary modes at the beginning and end of the

cycle are better confined to the edges.

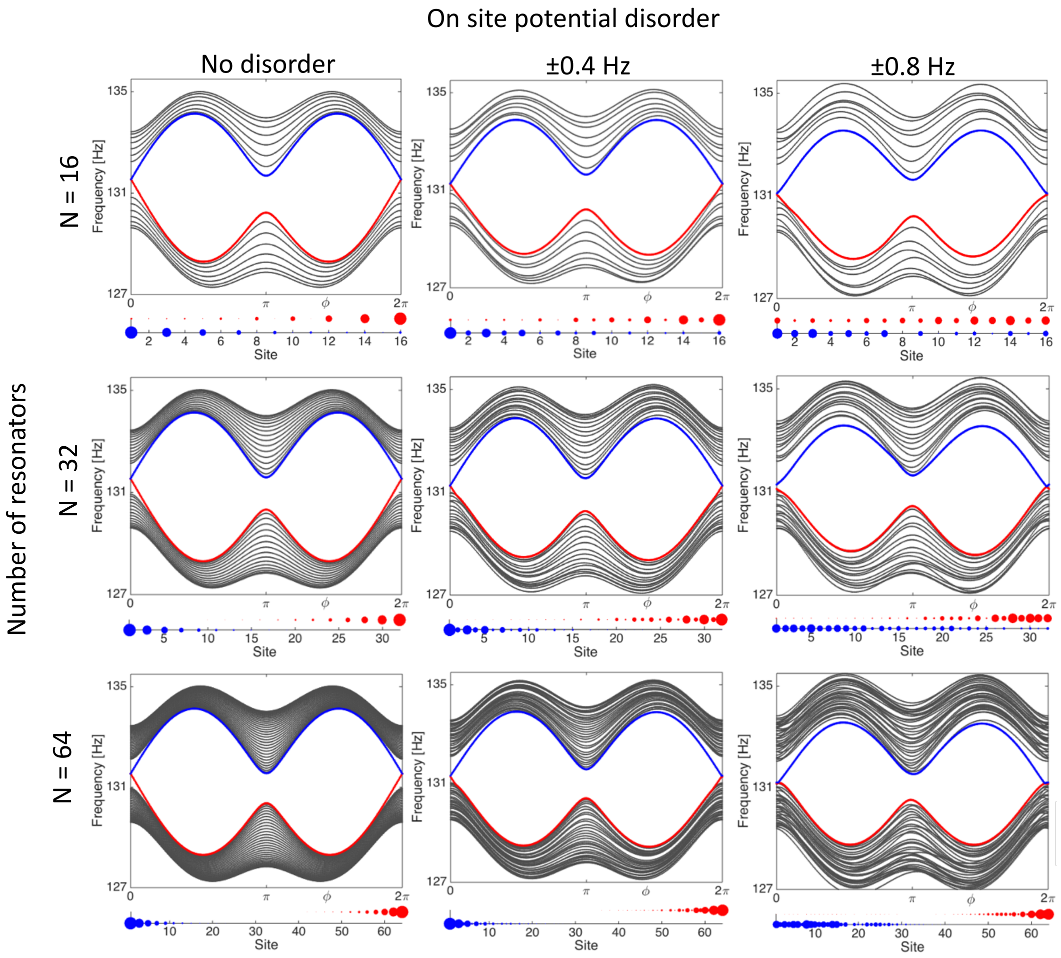

Supplementary Figure 12: Simulations of larger disordered arrays for (a) 16 resonators, (b) 32 resonators and (c) 64 resonators. The on-site potential disorder (i.e. shift of frequency) is added using a uniform random distribution corresponding to the column header. We present example pumping cycles with EM_L marked in red and EM_U marked in blue. Regardless of the strength of disorder, as long as the band gap remains open, the pumping cycle can always be completed provided we follow these modes adiabatically. Below each plot of band structure, we present the mode shape of $EM_{L,U}$ at $\phi = 0$ (we separate the modes slightly in the vertical direction only for clarity) with the corresponding red and blue colors. For a given length array, the disorder causes these modes to delocalize from their corresponding edges.

Supplementary Note 12

Experiments with ccw pumping

In the main text we presented experimental results of the measured transport fidelity values $F_{L\to R}, F_{L\to L}$, for the topological pump and the non-adiabatic process for clockwise (cw) rotation of the modulation shaft. Here we present experimental

results for ccw rotation of the modulation shaft (Supplementary Figure 13) which shows similar trends. The difference between cw and ccw rotations can be understood from the band-structure shown in Supplementary Figure 9a. For cw topological pumping we follow the lower edge mode EM_L, while for ccw pumping we follow the upper edge mode EM_U. Both yield the same outcome with the edge modes being transported from one side of the array to the other.

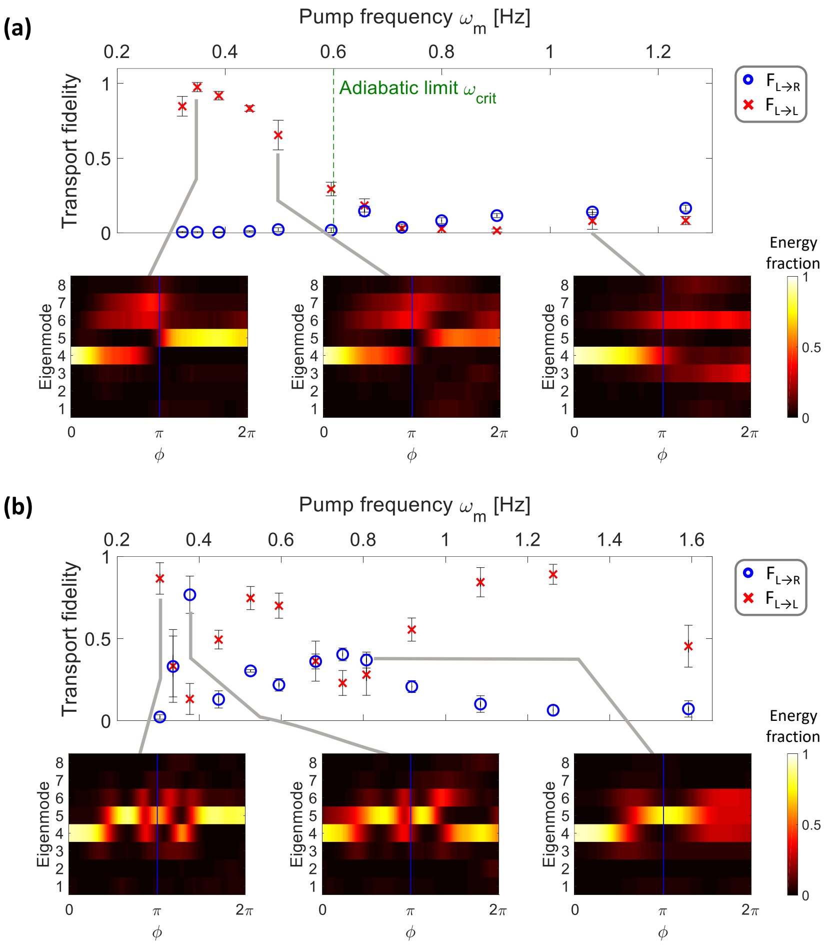

Supplementary Figure 13: Experimentally measured transport fidelity values for ccw rotation of the modulation shaft, for both (a) the topological pump (pumping along EM_U) and (b) the non-adiabatic process. Each data point is averaged over 10 consecutive measurements and the error bars represent the standard deviation amongst these measurements. The insets are examples of the modal energy fraction throughout the pumping cycle.

Supplementary Note 13

Experimental results for different defects

In this section we present additional experimental results that for the sake of brevity were not included in the main text.

In Supplementary Figure 14 we show the spatial distribution and centroid of EM_L throughout the pumping process for an unperturbed system.

Supplementary Figure 15 presents additional experimental results of the type shown in Figure 4 of the main text. In these experiments we incorporate static defects of on-site frequency detuning. We also include two examples where pumping ocurrs with low fidelity $F_{L\to R}$.

Supplementary Figure 16 and 17 present additional experimental results on temporal defects of the type shown in Figure 5a of the main text. Here we incorporate a temporal on-site frequency detuning for different sites in the array and at different angular positions of the modulation shaft.

Finally, in Supplementary Figure 18 we present a visual explanation of the phase boundary defect presented in Figure 5b of the main text.

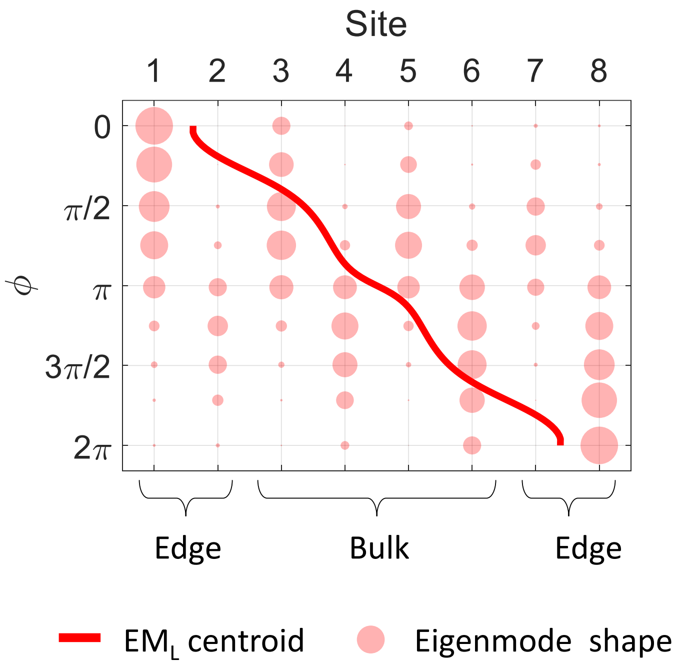

Supplementary Figure 14: Eigenvector spatial distribution for different values of ϕ in increments of $\pi/4$ are shown in shaded pink, where circle size corresponds to magnitude of the local excitation. The solid red line is the centroid of the eigenvector and helps visualize the spatial evolution of the mode across the array. This plot is for a system without any defects and is used as a visual aid to understand the spatio-temporal coordinates of the temporal defects in Supplementary Figure 16 and 17 and in Figure 5 in the main text.

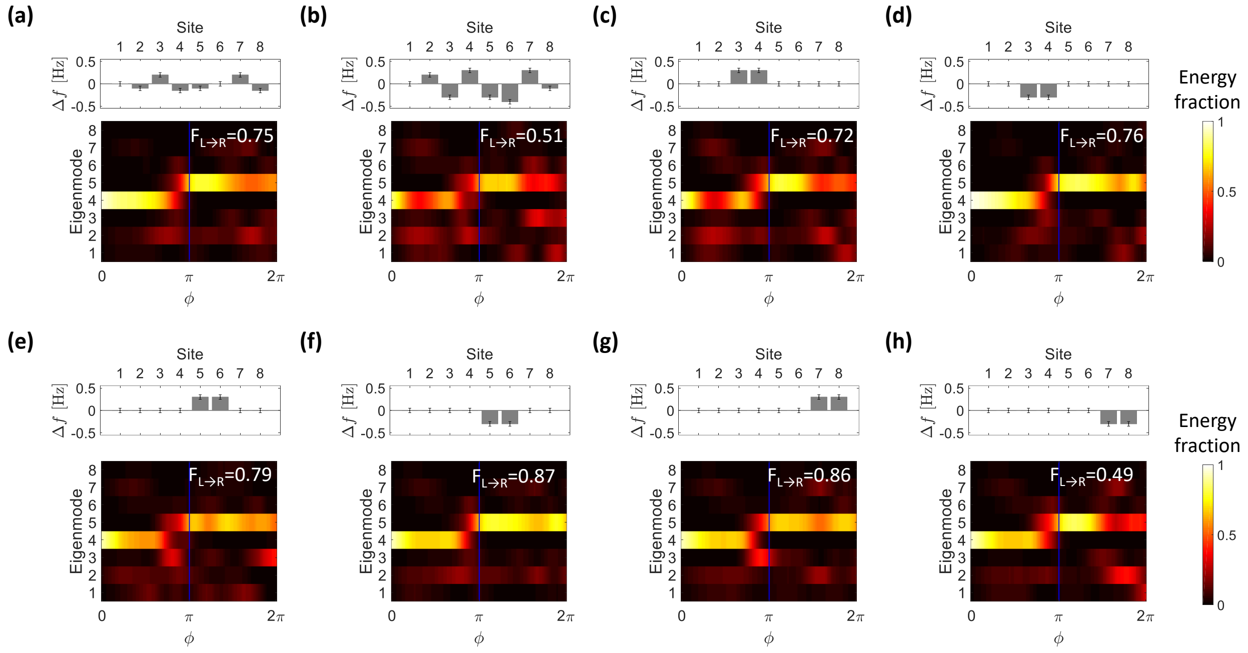

Supplementary Figure 15: Modal energy fraction for a system with spatial on site potential defects. The bar plots above each modal energy fraction indicates the amount of detuning applied at each site. All of the results are for modulation rate of $\omega_m \approx 0.3$ Hz. In (a) the resonance frequencies were randomly shifted in the range of ± 0.2 Hz which is $\sim 10\%$ of the band-gap. In (b) the resonance frequency of the array was randomly shifted in the range of ± 0.4 Hz which is $\sim 20\%$ of the system band-gap. In this case the array disorder is very large and the pump efficiency dropped to 50%. In (c-h) the resonance frequency of a single unit cell was shifted by either ± 0.3 Hz. When the defect is at the bulk unit cells (i.e. resonators 3-6) the transport fidelity remains high showing the system robustness.

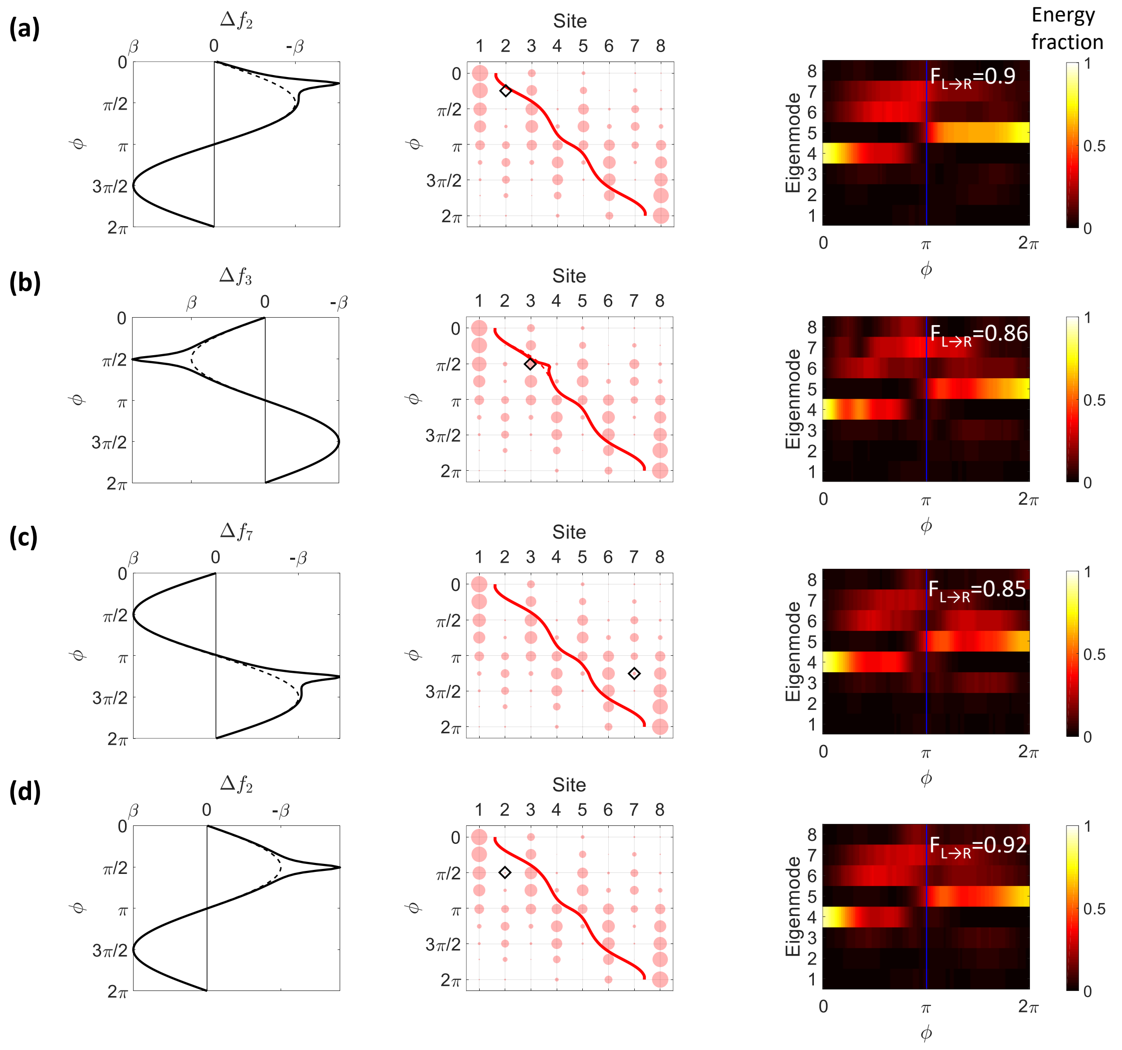

Supplementary Figure 16: Modal energy fraction throughout the pumping process for a system with different spatio-temporal defects of on-site potential, and modulation rate of $\omega_m \approx 0.3$ Hz. For each result we show schematically the evolution of the resonance frequency of the specific defect resonator $\Delta f_\#$. The simulated evolution of the $\mathrm{EM_L}$ eigenvector and centroid are also presented (see comparison to an unperturbed system in Supplementary Figure 14). The coordinates of the spatio-temporal defect are marked by a diamond on the centroid plot. Examples continue in Fig 17.

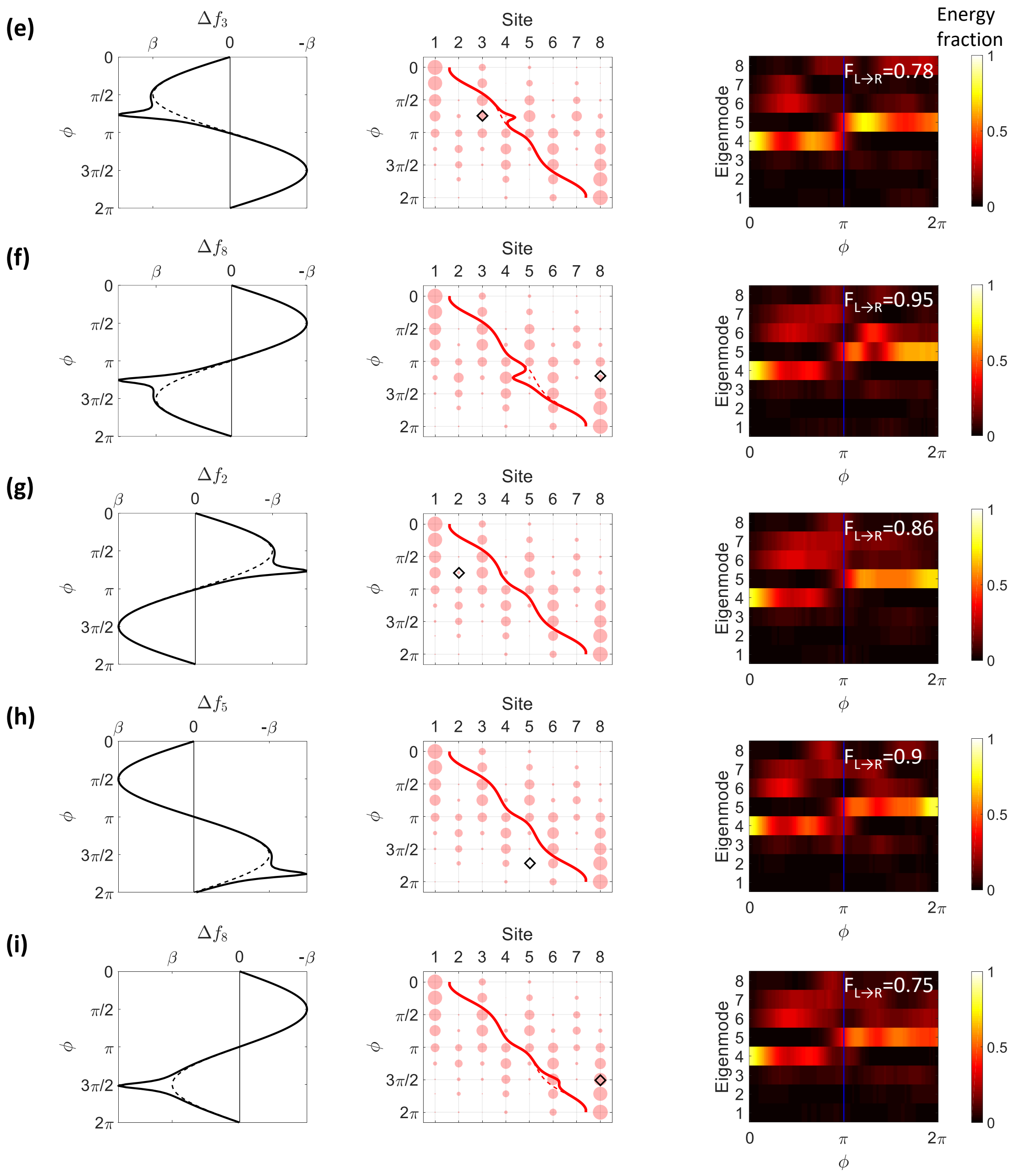

Supplementary Figure 17: Continuation of spatio-temporal defects from Supplementary Figure 16.

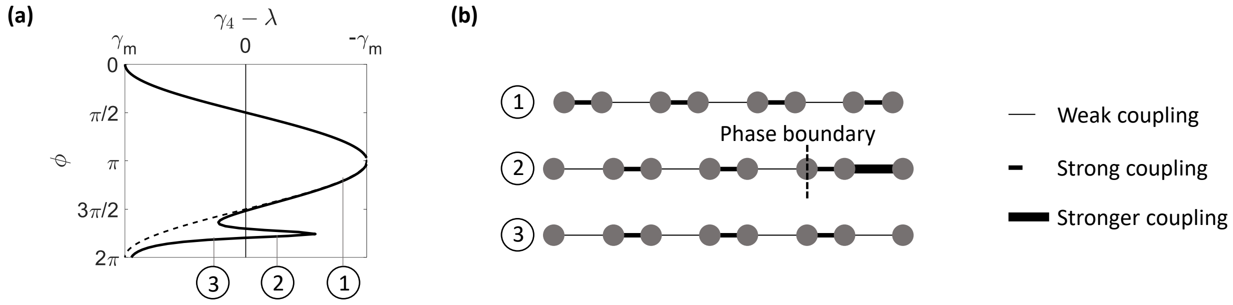

Supplementary Figure 18: A visual explanation of the phase boundary defect presented in Figure 5b of the main text. (a) The intracell coupling rate at the 4th unit cell was altered from the standard trajectory (as shown by the $\gamma_4 - \lambda$ plot) near the end of the pump cycle. Three points of interest are identified for closer examination, indicated by (1), (2), and (3). (b) At these points, we visualize the tight-binding form (ignoring on-site potentials) with line width corresponding to the coupling strength. At point (2) the system exhibits the intrusion of a phase boundary between trivial and topological phases.

References

[1] Landecker, P. B., Villani, D. D. & Yung, K. W. An analytic solution for the torque between two magnetic dipoles. *Physical Separation in Science and Engineering* **10**, 29–33 (1999).

[2] Grinberg, I. H. *et al.* Magnetostatic spring softening and stiffening in magneto-mechanical resonator systems. *IEEE Transactions on Magnetics* **55**, 4003505 (2019).

[3] Griffiths, D. J. & Schroeter, D. F. *Introduction to Quantum Mechanics* (Cambridge University Press, 2018).

[4] Born, M. & Fock, V. Beweis des adiabatensatzes. *Zeitschrift für Physik* **51**, 165–180 (1928).
