# Supplementary material for: Robust temporal pumping in a magneto-mechanical topological insulator
Source: Nat Commun. 2020 Feb 20;11:974. doi: 10.1038/s41467-020-14804-0 (PMC7033202; doi:10.1038/s41467-020-14804-0)
Supplement: Supplementary file 2 — Description of Additional Supplementary Files [file 41467_2020_14804_MOESM2_ESM.pdf]

Description of Additional Supplementary Files

Supplementary Movie 1

This animation shows a single unit cell of the magneto-mechanical array during the pumping cycle from 0 to 2\pi. The two resonators in the unit cell are marked as Site A and Site B. Angular rotation of the modulation shaft induces on-site potential modulation (OPM) via permanent magnets (shown as blue and red disks), as well as coupling modulation through the off-axis high-permeability metal alloy sheet (shown in green).
